# Systematic identification of interaction effects between genome- and environment-wide associations in type 2 diabetes mellitus

**DOI:** 10.1007/s00439-012-1258-z

**Published:** 2013-01-20

**Authors:** Chirag J. Patel, Rong Chen, Keiichi Kodama, John P. A. Ioannidis, Atul J. Butte

**Affiliations:** 1 Division of Systems Medicine, Department of Pediatrics, Stanford University School of Medicine, 1265 Welch Road, Room X-163 MS-5415, Stanford, CA 94305 USA; 2 Lucile Packard Children’s Hospital, Palo Alto, CA 94304 USA; 3 Stanford Prevention Research Center, Department of Medicine, and Department of Health Research and Policy, Stanford University School of Medicine, Stanford, CA 94305 USA

## Abstract

Diseases such as type 2 diabetes (T2D) result from environmental and genetic factors, and risk varies considerably in the population. T2D-related genetic loci discovered to date explain only a small portion of the T2D heritability. Some heritability may be due to gene–environment interactions. However, documenting these interactions has been difficult due to low availability of concurrent genetic and environmental measures, selection bias, and challenges in controlling for multiple hypothesis testing. Through genome-wide association studies (GWAS), investigators have identified over 90 single nucleotide polymorphisms (SNPs) associated to T2D. Using a method analogous to GWAS [environment-wide association study (EWAS)], we found five environmental factors associated with the disease. By focusing on risk factors that emerge from GWAS and EWAS, it is possible to overcome difficulties in uncovering gene–environment interactions. Using data from the National Health and Nutrition Examination Survey (NHANES), we screened 18 SNPs and 5 serum-based environmental factors for interaction in association to T2D. We controlled for multiple hypotheses using false discovery rate (FDR) and Bonferroni correction and found four interactions with FDR <20 %. The interaction between rs13266634 (SLC30A8) and trans-β-carotene withstood Bonferroni correction (corrected *p* = 0.006, FDR <1.5 %). The per-risk-allele effect sizes in subjects with low levels of trans-β-carotene were 40 % greater than the marginal effect size [odds ratio (OR) 1.8, 95 % CI 1.3–2.6]. We hypothesize that impaired function driven by rs13266634 increases T2D risk when combined with serum levels of nutrients. Unbiased consideration of environmental and genetic factors may help identify larger and more relevant effect sizes for disease associations.

**Electronic supplementary material:**

The online version of this article (doi:10.1007/s00439-012-1258-z) contains supplementary material, which is available to authorized users.

## Introduction

Complex diseases like type 2 diabetes (T2D) have multifactorial etiologies, with genetic and environmental factors playing roles (Schwartz and Collins 2007). Genome-wide association studies (GWAS) have identified many common single nucleotide polymorphisms (SNPs) associated with disease (Hindorff et al. 2009b; Visscher et al. 2012). However, individual SNPs confer modest risks, and cumulatively, they account for only a limited portion of missing heritability (Manolio et al. 2009), and they have little prognostic utility (Meigs et al. 2008). Furthermore, it has been reported that the availability of entire genomes may not be clinically informative (Roberts et al. 2012). As a result, many models have been proposed or re-introduced to describe the genetic basis of complex disease (Gibson 2011; McClellan and King 2010).

Gene–environment interactions have been an important concept in evolutionary biology. For example, gene–environment interactions result in phenotypic plasticity, where a given genotype produces different phenotypes in response to different environmental conditions. The spectrum of possible phenotypes, such as variation in disease risk, is known as the “reaction norm” (Pigliucci 2001). Thus, interactions may account for some of unexplained disease risk and/or improve our understanding of genetic basis of risk. In human disease epidemiology, gene–environment interactions describe the effect size of the combination of genetic and environmental factors as different than the effects of each factor alone (Hunter 2005; Thomas 2010). In the following, we consider this type of statistical gene–environment interaction.

Statistical interaction may offer hints about biological interaction, where genetic and environmental factors jointly determine physiological effects on a molecular or cellular level (Wang et al. 2010). Biological interactions may be informative of disease etiology. For example, genetic risk for bladder cancer associated with variants of the *NAT2* gene, a gene that plays a possible role in metabolism of tobacco smoke constituents, is known to be larger when considering smoking status of individuals (Rothman et al. 2010). Thus, identification of robust statistical/epidemiological interactions can be seen as a first step toward creation of hypotheses relevant to disease pathophysiology.

We have created a method called environment-wide association study (EWAS). EWAS finds environmental factors associated with disease (Patel et al. 2010, 2012b; Tzoulaki et al. 2012). EWAS is analogous to GWAS in which it evaluates multiple environmental factors and has proper adjustment for the multiplicity of comparisons. The associations that emerge are validated across different datasets. In sum, EWAS is a way to unify differences between genetic and environmental association studies and to accelerate our knowledge regarding potential environmental influences on health and disease (Ioannidis et al. 2009).

Most studies in this area examine genetic or environmental factors. However, it is still rare for both types of factors to be measured in human tissue concurrently. A key challenge involves choosing which factors to examine. Analyzing all of them is not possible with current technology (Thomas 2010), and factors are often selected for convenience, without sufficient documentation of the strength of their marginal associations. Given the complexity of gene–environment interaction analyses, there may be problems with selective analyses and selective reporting of results in a fragmented and possibly biased fashion (Ioannidis 2005). For example, many studies do not account for all the interaction effects that they explore. Thus, there is a need to select common SNPs and exposures and systematically screen their interactions to avoid spurious results (Khoury and Wacholder 2009; Patel et al. 2012a).

Here, we used a data-driven and systematic approach for selecting gene–environment interactions associated with a common disease such as T2D. Our specific goal was to test NHANES data (Centers for Disease Control and Prevention (CDC) 2009) for interactions between robust factors found in GWAS and EWAS. NHANES includes 261 genotyped loci, 266 environmental factors measured in blood and urine, and clinical measures for the same individuals. We focused on the top GWAS and EWAS hits, and systematically investigated SNP–environment interactions associated with T2D. Top GWAS hits were defined as SNPs that had been associated with T2D in at least one study. Top EWAS hits had robust associations and low false discovery rates (FDRs) in multiple cohorts. In our statistical specification of interactions, we modeled disease risk due to the combination of genotypic and environmental factors as different than the sum of the risks of each factor alone (Khoury et al. 1988; Thomas 2010).

A source of major debate for the etiology of T2D is the thrifty genotype hypothesis, in which thrifty genes provided advantages to human populations during the hunter–gatherer era (Diamond 2003; Neel 1962; Zimmet et al. 2001). People with the thrifty genotype stored food energy efficiently during times of feast, making more available to them during times of famine (Neel 1962; Zimmet et al. 2001). Thus, in modern societies, thrifty genotypes may have become risk genotypes. However, evidence to support this hypothesis is lacking, and competing hypotheses have emerged. A data-driven study of interactions between SNPs and common environmental exposures may shed light on this debate and to bring to fore its clinical implications.

## Materials and methods

### Data and selected genetic and environmental factors

Data came from National Health and Nutrition Examination Survey (NHANES) (Centers for Disease Control and Prevention (CDC) 2009). All SNPs available had been chosen a priori by independent researchers investigating other topics (Matise et al. 2011). These SNPs were assayed in two NHANES: 1999–2000 and 2001–2002. Genotypes were not collected in 2003–2004 and 2005–2006 and have yet to be released for 2007–2008. Of these, we used 18 SNPs with documented near-GWS associations in T2D (for examples and references, see Table S1). We computed allele frequencies of each SNP stratified on race to confirm their presence. For quality control, we estimated deviation from Hardy–Weinberg equilibrium (HWE) for each SNP by race. In NHANES, ethnicity was coded in five groups (Mexican-American, non-Hispanic black, non-Hispanic white, other Hispanic, other).

We previously used NHANES data from 1999–2000, 2001–2002, 2003–2004, and 2005–2006 to screen 266 environmental factors measured in blood or urine (Patel et al. 2010). We identified and tentatively validated five environmental factors associated with T2D: trans-β-carotene, cis-β-carotene, γ-tocopherol, heptachlor epoxide, and PCB170. The FDR for each association was <10 % in at least 2 independent surveys. Trans- and cis-β-carotenes were measured in the 2001–2002, 2003–2004, and 2005–2006 surveys and had a significance threshold under FDR 10 % for all 3 surveys. γ-Tocopherol was measured in all four surveys. Its FDR was <10 % in 1999–2000, 2003–2004, and 2005–2006 and <20 % in the 2001–2002 survey. Heptachlor epoxide was measured in the 1999–2000, 2001–2002, and 2003–2004 surveys. Its FDR was <10 % in the 1999–2000 and 2003–2004 surveys. PCB170 was measured in the 1999–2000, 2001–2002, and 2003–2004 surveys and its FDR was <10 % in the 1999–2000 and 2003–2004 surveys. In the current investigation, we analyzed the 1999–2000 and 2001–2002 surveys.

T2D was defined in survey participants having 8.5-h fasting blood glucose (FBG) values ≥126 mg/dL, as advised by the American Diabetes Association. We acknowledge neither FBG nor the self-reported diabetes status distinguishes between Type 1 diabetes (T1D) and T2D, but given that T2D accounts for over 95 % of all diabetes cases, we assume most of our cases are T2D. To increase study power, we combined data from the 1999–2000 and 2001–2002 surveys. Depending on the genetic and environmental variables tested, there were 841–2,655 controls and 81–274 case subjects.

Each SNP was coded for the number of risk alleles as designated in the publications citing it (Chen et al. 2010). Environmental factors were continuous and followed a long-tailed distribution; thus, they were log-transformed and standardized (expressed in standard deviation units) (Patel et al. 2010).

We compared age, BMI, sex distribution, and race in T2D subjects and controls. Risk alleles of SNPs from the literature were found and their frequencies in NHANES subjects were computed by race. We computed baseline levels of environmental factors in both groups. We assessed the marginal effect between SNPs or environmental factors on T2D with survey-weighted logistic regression, adjusting for race, age, sex, and BMI. We also assessed marginal effects stratified by race.

### Baseline characteristics for each subsample

We defined a subsample as a group of survey participants available for each interaction test. SNPs and environmental factors were not assayed equally among subjects, so each subsample contained a different number of participants.

We computed baseline characteristics for each subsample to assess overall differences between them. First, we computed risk allele frequency stratified by race in the HapMap as follows: CEU (Americans of European decent living in Utah), ASW (Americans of African ancestry living in the Southwest), and MEX (Americans of Mexican ancestry living in Southern California) (Frazer et al. 2007).

We ascertained whether SNPs might be correlated with serum levels of environmental factors. We evaluated the correlation between genetic and environmental factors through survey-weighted linear regression, regressing log base 10 of the environmental exposure variable on each genetic variable, adjusted for race, sex, age, and BMI.

### Systematic interaction screen between SNPs and environmental factors

**Fig. 1:**
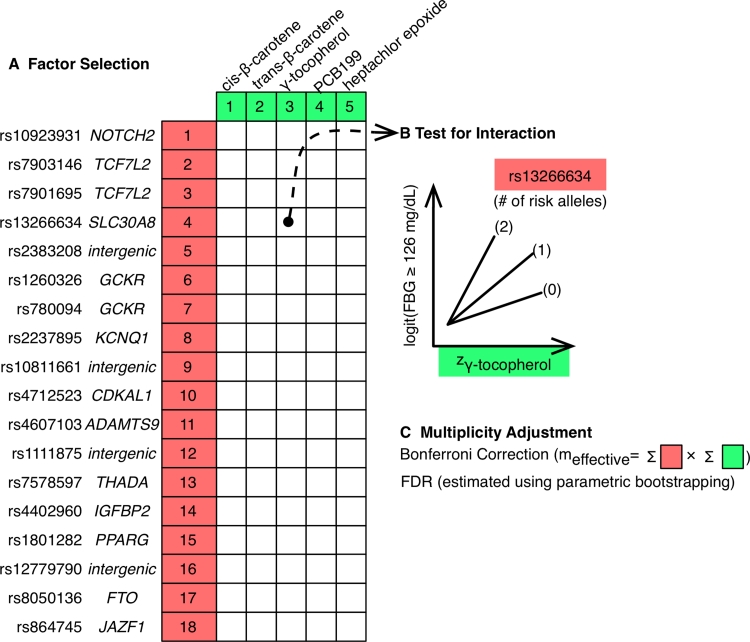
Comprehensive testing and screening for gene-environment interactions in T2D. **a** Genetic and environmental factors were chosen by their strength of marginal association in GWAS and EWAS; **b** each SNP/exposure pair was tested for interaction in association to disease in a logistic regression model adjusting for other risk factors and main effects of exposure and SNP; **c** we used a modified Bonferroni correction to control for multiple hypotheses were and the FDR was estimated. *FDR* false discovery rate

Next, we screened the 90 possible pairs for interaction (18 genetic loci times 5 environmental factors; Fig. 1a, b). We utilized survey logistic regression to associate each pair of factors to T2D, incorporating a multiplicative interaction term and main effects of both factors. Each model was adjusted by age, sex, race, and BMI.

NHANES is a complex multi-staged survey and we accounted for its sampling methodologies as recommended by the National Centers for Health Statistics (NCHS) (CDC and National Center for Health Statistics (NCHS) 2003). Specifically, we used 4-year survey probability weights corresponding to the smallest subsample analyzed to accurately estimate point estimates of effects (Vittinghoff et al. 2005). We also accounted for clustering and stratification of the survey to compute standard errors and *p* values accurately (Vittinghoff et al. 2005). We used SAS version 9.2 and the “PROC SURVEY” suite of commands, as also recommended by the NCHS (CDC and National Center for Health Statistics (NCHS) (2003). Restricted data were accessed with permission through a Research Data Center (RDC) in Hyattsville, Maryland.

### Multiplicity correction and false discovery rate estimation

Bonferroni multiplicity correction adjusts the threshold for statistical significance by the number of statistical tests conducted. Since our tests were not independent, we estimated the total number of effective genetic loci and environmental exposures tested jointly by accounting for the correlation between selected factors. This approach estimates the number of hypotheses for a group of correlated factors and has been applied to the study of SNPs (Nyholt 2004). We expanded the method to environmental factors. For the 18 SNPs, we calculated the correlation between SNPs stratified by race, and concluded that there were 17.7 effective genetic factors. For the 5 environmental factors, we calculated 4.41 effective factors. Thus, the total number of effective tests was 78.1 (17.7 × 4.41). The adjusted level of significance for a single test with a threshold of *p* = 0.05 was 0.0006 (0.05/78.1).

We also calculated the FDR (Storey 2002), the expected ratio of the number of false positives to the total number of positives, or the expected fraction of results drawn from the null distribution at a given significance level (Benjamini and Hochberg 1995). To estimate the number of false positives, we generated a distribution of null test statistics corresponding to an interaction term, while preserving the main effects of the SNP and exposure terms, using a parametric bootstrap method (Bůžková et al. 2011).

The parametric bootstrap methodology is a randomization technique that simulates the distribution of test statistics for the interaction term under the null hypothesis of no interaction. To create the null distribution of test statistics corresponding to the interaction term (*β*_*G*×*E*_), we fit a logistic regression model omitting the interaction term (*β*_*G*×*E*_ = 0), while leaving parameters that modeled the main effects of the environmental factor, SNP, and age, sex, race, and BMI. We “bootstrapped” (randomized with replacement) fitted dependent values (predicted odds for diabetes) from the null model and refit the interaction model described above, adding the covariate corresponding to the interaction between SNP and environmental factor (*β*_*G*×*E*_). To simulate a null distribution of test statistics, this procedure was repeated 100 times. The FDR was estimated as the ratio of interaction terms deemed significant in the simulated distribution to all results deemed significant in the real data.

The specific bootstrapping procedure was as follows:

1. We screened all 90 interaction pairs and collected all *p* values corresponding to the coefficient of the interaction, *β*_*g*×*e*_. We called these *p* values *P*_real_. These models were specified as:

$${\text{Logit}}\left( {{\text{T}}2{\text{D}}} \right) = \alpha + {\beta_{\text{g}}}*{\text{g}} + {\beta_{\text{e}}}*{\text{e}} + {\beta_{\text{gxe}}}*{\text{g}}*{\text{e}} + \left( {{\text{other}}\,{\text{covariates}}} \right)\,\,\left( {{\text{Model}}\, 1} \right)$$

Here, “*g*” denotes the SNP coded by the number of risk alleles and “*e*” is the environmental factor, mean-centered and standardized by the standard deviation.

2. For each of the 90 interaction pairs, we computed the model corresponding to “no interaction,” or *β*_*g*×*e*_ = 0:

$${\text{Logit}}\left( {{\text{T}}2{\text{D}}} \right) = \alpha + {\beta_g} \times g + {\beta_g} \times e + \left( {{\text{other}}\,{\text{covariates}}} \right)\,\,\left( {{\text{Model}}\, 2} \right) $$

The predicted values for Logit(T2D) are called *Y*.

3. From Model 2 corresponding to *β*_*g*×*e*_ = 0, we bootstrapped sample (sample with replacement) *Y*. They were used as dependent values for Model 1, which corresponded to the interaction model. We collected *p* values corresponding to the interaction term *β*_*g*×*e*_.
4. Steps 2 and 3 were repeated 100 times, leading to 100 × 90 (9,000) models. The set of 9,000 *p* values collected in this way was called *P*_null_.
5. We estimated the FDR for a given significance level. For example, for a significance level of 0.05:

FDR(0.05) = ((#*P*_null_ < 0.05)/100)/(#*P*_real_ < 0.05).

### Power calculations for interactions

Sample sizes, genetic risk allele frequency and marginal OR, and environmental exposure marginal OR were used to compute power to detect moderate-to-high interaction effects (interaction OR = 1.5 and 2.0) at *p* value of 0.01 (FDR <18 %). We assumed marginal effects of genetic factors observed among NHANES cohorts (genetic marginal OR = 1.0) and environmental factors OR as observed in EWAS (exposure marginal OR = 1.5). We used *Quanto* software for these calculations (Gauderman and Morrison 2009).

### Analyses stratified by race and consideration of other T2D risk factors

Our main analysis scan included all participants from diverse ancestral groups, as reflected in NHANES. Given that the strongest evidence for T2D associations has come from studies of Caucasians, we also performed interaction analyses stratified by race.

We investigated whether eight other candidate T2D risk factors interact with 18 SNPs. These other factors included BMI and pulse rate (a proxy for physical fitness). They also included self-reported intake variables derived from a 24-h dietary questionnaire. In this questionnaire, NHANES participants were queried regarding foods they consumed in 24 h prior to the survey. Variables derived from this questionnaire include total energy (as calories), carbohydrate, monounsaturated fat, polyunsaturated fat, total saturated fat, and total fat consumed in 24 h. As above, all variables were standardized by the population mean and standard deviation. Interaction models were specified as above (main effects and multiplicative interaction term), adjusting for BMI, self-reported race, sex, and age. Sample sizes for these tests were greater than in our main analyses (*N* = 278–291 cases, 3,066–3,139 controls).

## Results

### Methodological overview

Figure 1 shows a schematic overview of our approach to search for interacting SNPs and environmental factors associated with T2D (defined as fasting blood glucose ≥126 mg/dL as recommended by the American Diabetes Association). We used a dataset containing measurements for SNPs and environmental factors and chose data with strong evidence of association of marginal effects in GWAS and EWAS.

GWAS provide a framework for assembling robustly replicated sets of common SNPs with genome-wide significance [GWS, *p* < 5 × 10^−8^ (Pearson and Manolio 2008)]. As noted above, EWAS provides a way to search for and validate environmental factors associated with disease (Patel et al. 2010). We selected environmental factors with significant associations in 2–4 independent surveys after accounting for the multiplicity of analyses and adjusting for demographic factors.

First, we examined the separate marginal effects of each SNP/genetic variant (“*G*”) or each environmental factor (“*E*”) on T2D. Next, we computed correlations between each environmental factor and SNP (total of *E* × *G* correlations) to ascertain the degree of their dependence.

Each environmental factor and SNP (total of *E* × *G* statistical tests) was tested for interaction while adjusting for age, sex, BMI, and race (self-reported) (Fig. 1b) in association to T2D. Finally, we accounted for multiplicity of analyses with both Bonferroni-adjusted *p* values and FDR estimation (Fig. 1c).

### Baseline characteristics of cohorts

Table 1 shows that the mean ages of diabetics and non-diabetics who participated in the 1999–2002 surveys differed (56 years for diabetic vs. 40 years for non-diabetics), as did their sex (39 % female diabetic vs. 52 % female non-diabetic), and mean BMI (32 vs. 27 kg/m^2^).

**Table 1:** Baseline differences between type 2 diabetics (fasting blood glucose ≥126 mg/dL) and non-diabetics for participants in the 1999–2002 NHANES

	Diabetics (FBG ≥126 mg/dL)		Controls (FBG <126 mg/dL)		*N* (diabetics)
	Mean	95 % CI	Mean	95 % CI	
Age	55.7	52.9–58.4	39.9	38.8–40.9	6,476 (456)
Female	39 %		52 %		6,476 (456)
Body mass index (kg/m^2^)	31.9	30.4–33.3	26.9	26.6–27.1	6,332 (436)
Race					
Non-Hispanic white	66 %	57–74	70 %	66–75	6,476 (456)
Mexican	7 %	5–9	7 %	5–9	
Black	12 %	8–14	11 %	9–16	
Other Hispanic	8 %	3–10	7 %	1–16	
Other	7 %	3–6	5 %	1–13	

### Risk allele frequency and genetic marginal associations

Figure S1 shows estimated allele frequencies and main effects of the risk allele for each of the 18 SNPs in the two NHANES. Risk allele frequencies were >5 % (or <95 %) for all of the ethnicities, except for rs1801282 (97 % risk allele frequency/3 % minor allele frequency) in blacks (Figure S1). We estimated deviation from HWE per race through a Chi-squared test. Gross deviations from HWE were not found in the Mexican-American, non-Hispanic white, and non-Hispanic black races (*p* > 0.05).

Three of 18 SNPs were marginally associated with T2D at significance level of 0.05 after adjustment for age, sex, race, and BMI. They were rs10923931 (*NOTCH2*), rs7903146 (*TCF7L2*), and rs13266634 (*SLC30A8*) (Figure S1). These data are uncorrected for multiple hypotheses, given that these SNPs have been associated with T2D in populations of European descent (Table S1).

We computed the marginal effect sizes for each SNP in non-Hispanic whites, non-Hispanic blacks, and Mexican-Americans to assess the potential effects of race on the marginal estimates (Figure S1). We did not find any strong associations for most of the 18 loci (uncorrected *p* > 0.05 for most SNPs for all races). Notable exceptions were rs13266634 (*SLC30A8*, *p* = 0.05 for non-Hispanic white), rs2237895 (*KCNQ1*, *p* = 0.04 for non-Hispanic black), rs8050136 (*FTO*, *p* = 0.001 for Mexican-Americans), rs7903196 (*TCF7L2*, *p* = 0.02 for non-Hispanic white).

SNPs nominally (*p* < 0.05) or marginally (*p* < 0.15) associated with T2D had OR similar to that observed in the literature (Table S1). However, marginal effects of other SNPs were null (OR 1.0–1.1), to be expected given our smaller cohorts (NHANES) and small effects observed in GWAS (Table S1).

### Environmental factor marginal associations

We had detected five environmental factors in our T2D EWAS (Patel et al. 2010). Figure S2 shows marginal T2D associations between diabetics and non-diabetics and average serum levels of these factors. The pollutant factors PCB170 and heptachlor epoxide had adjusted odds ratio of 1.7 and 1.5, respectively (*p* = 0.01, adjusted for age, sex, race, and BMI) for a 1 SD change in logged exposure. The nutrient factor γ-tocopherol had an adjusted odds ratio of 1.5 (*p* < 0.001) and two β-carotene factors were associated with protection from T2D, with odds ratios of 0.6 (*p* = 0.003 and 0.006). While we observed some differences in mean serum levels of environmental factors between races, the directions of effect size estimates were consistent between them (Figure S2).

To study interactions between genotypes and environmental factors, we computed the sample sizes available for each pair of environmental factor and SNP, defined here as “subsamples.” Subsamples contained 81–274 diabetic case participants and 841–2,655 control participants. In spite of the differences in absolute numbers, the percentage of diabetics per subsample was constant at 9–10 % (Figure S3A).

Power calculations for genotype–environment interactions depend on minor allele frequency, environmental factor variability, the ratio of cases to controls, and marginal associations to disease (Gauderman and Morrison 2009). We estimated the minor allele frequencies in our NHANES participants (5–44 %), the ratio of cases and controls for each genotype–environment factor pair (8–10 %), and assumed standardized environmental variables (SD = 1). Further, we assumed a genetic marginal OR of 1.0 and environmental factor OR of 1.5 as observed in these NHANES participants (Figure S1, S2). Under these assumptions, we determined power of 50–100 % (median 93 %) to detect an interaction OR of 2.0 for a significance threshold *α* = 0.01 (corresponding to FDR <20 %) (Gauderman and Morrison 2009) (Figure S3B).

### Subsample characteristics

Figure S4A, B shows our estimates of the per-subsample risk allele frequency and estimates of the genetic marginal effect size. Overall, we found few differences in risk allele frequency between subsamples by race (Figure S4A). Further, we found little difference between risk allele frequency between non-Hispanic whites, Mexican-Americans, and non-Hispanic blacks compared to the CEU, MEX, and ASW HapMap populations (Frazer et al. 2007) (Figure S4A).

### Correlations between SNPs and environmental factors

We found little correlation between the 18 SNPs and the 5 environmental factors (Figure S5A–E). There was a nominal negative association between rs10923931 and heptachlor epoxide (*p* = 0.02), where levels of heptachlor epoxide decreased 10 % per risk allele. We also observed a negative association between rs10923931 and cis-β-carotene (*p* = 0.04), where levels of heptachlor epoxide decreased 5 % per risk allele.

### Correlations among environmental factors

We computed the pair-wise correlations between each of the environmental factors (Table S2) to assess their interdependency. There was moderate-to-low correlation between most factors (*ρ* ranging from −0.2 to 0.34). For example, there was a low inverse correlation between both versions of β-carotene and γ-tocopherol (*ρ* = −0.2); however, we observed high correlation between cis-β-carotene and trans-β-carotene (*ρ* = 0.96).

### Screening for SNP by environment interactions

**Fig. 2:**
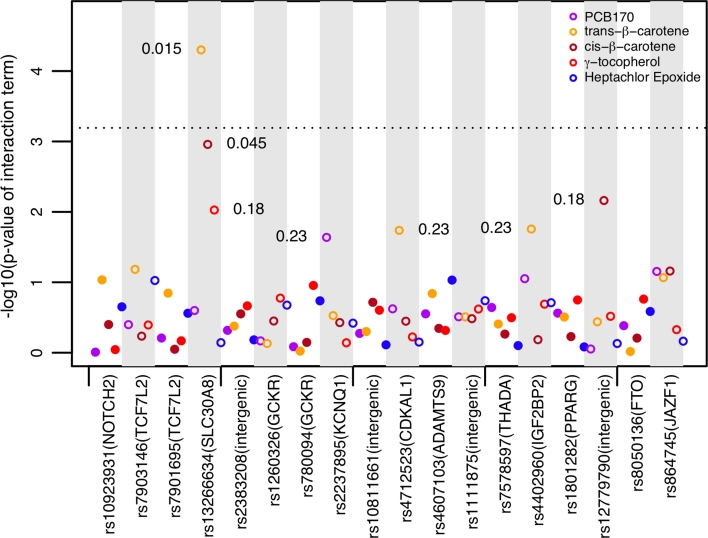
Significance values of interaction term [−log10(*p* value) for interaction term of pair of factors]. SNPs are on the *x* axis and environmental factors are color-coded above each SNP. Markers alternate between filled and open for each locus. The *y* axis shows −log10(*p* value). Interactions with *p* value ≤0.05 are annotated with their FDR. The *dotted line* denotes the Bonferroni threshold. One finding (the interaction between rs1326634 and trans-β-carotene; FDR = 1.5 %) was *above the line*

To study interactions between the 18 SNPs and the 5 environmental factors, we tested 90 interactions using survey-weighted logistic regression adjusted for age, sex, race, and BMI. Figure 2 is a Manhattan-style plot showing results for the 90 interaction terms. Seven results (5 SNPs and 4 environmental factors) had *p* values ≤0.05 (FDR ≤23 %). FDRs for these seven results were between 1.5 and 23 %. We discuss interactions that have reached FDR <25 % here.

**Fig. 3:**
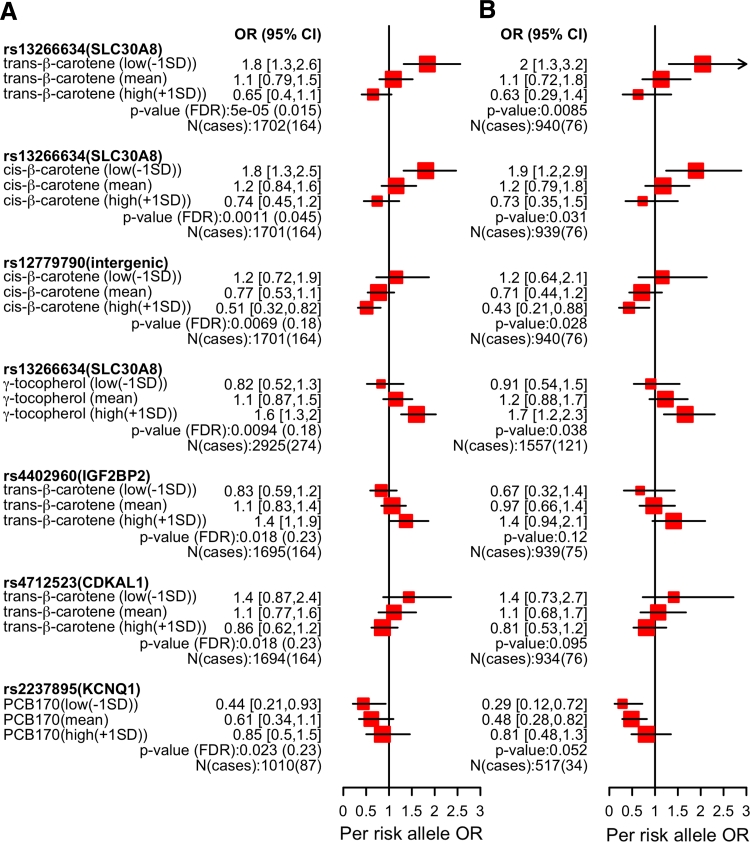
Per-risk allele effect sizes for top putative interactions with *p* < 0.05. **a** Estimates for all races, **b** non-Hispanic whites. *Markers* denote interaction OR computed at 1SD below mean exposure levels, at the mean, or at 1 SD greater than the mean. Marker sizes are proportional to inverse variance

Our top interaction was between trans-β-carotene (a nutrient marker) and the non-synonymous SNP rs13266634 (*SLC30A8*). Specifically, higher levels (defined as 1 SD above the mean) of this nutrient factor appeared to have a protective effect. The interaction was significant beyond the Bonferroni-adjusted cutoff level (interaction *p* = 5 × 10^−5^, Bonferroni adjusted *p* value 0.006, FDR = 1.5 %). At lower levels of trans-β-carotene (defined as 1 SD below the mean), the per-allele effect size/OR was 1.8 (95 % CI 1.3–2.6), which was 40 % greater than the marginal effect (Fig. 3a). We also estimated the environmental risk for the different genotypes. For example, the adjusted OR per change in trans-β-carotene levels (environmental effect size) was protective in subjects with two risk alleles for the SNP (adjusted OR 0.5, 95 % CI 0.4–0.7), while the effects were negligible in subjects with ≤1 risk alleles (Figure S6), suggesting protective effects of trans-β-carotene for individuals with two risk alleles. We observed similar effects for cis-β-carotene and rs13266634 (Fig. 3a).

We observed an opposite effect in subjects with the rs13266634 risk alleles as levels of γ-tocopherol rose (interaction *p* = 0.009, FDR = 18 %, Fig. 3a); γ-tocopherol had an adverse effect in combination with the rs13266634 risk genotype. When γ-tocopherol levels were 1 SD higher than the mean, the adjusted OR was 1.6 (adjusted 95 % CI 1.3–2), a 25 % increase in per-allele adjusted OR when compared to the marginal effect (Figure S1). Genetic risk in subjects below the mean levels of γ-tocopherol appeared mitigated.

We did not detect a marginal individual association between intergenic SNP rs12779790 and T2D, but we did observe an interaction with this locus and trans-β-carotene (Fig. 3a). The protective effect of trans-β-carotene increased 50 % in subjects with two risk alleles, compared to 0.6 for its marginal per-SD effect (Figure S6). This was an adjusted per-SD environmental factor OR of 0.3 (95 % CI 0.2–0.5).

Similarly, we did not detect a marginal association between rs2237895 (*KCNQ*) and T2D, but we saw environmental risk of PCB170 equal to 2.8 (95 % CI 1.7–4.5, Figure S6) for individuals with two risk alleles. This was twofold greater than the marginal estimate of 1.7 (Figure S2).

### Interactions stratified by race

We computed interaction estimates separately for each race. As expected, estimates for non-Hispanic whites were comparable to those of all participants (Fig. 3a, b). However, the study was underpowered for observing interaction effect sizes in Mexican-Americans or non-Hispanic blacks (Figure S7A, B). We observed amplified effects for some per-race stratified interactions. For example, non-Hispanic blacks with low levels of cis-β-carotene and risk alleles for rs12779790 had a threefold increased risk for T2D compared to the overall sample (Figure S7A). Similarly, Mexican-Americans with low levels of trans-β-carotene and risk alleles for rs4712523 (*CDKAL1*) also had twofold increased risk for T2D compared to all participants (Figure S7B).

### Interactions adjusted by cis-β-carotene

The interaction OR conferred by the combination of cis/trans-β-carotene and rs13266634 was similar and in the same direction (Fig. 3). Furthermore, the interaction OR between γ-tocopherol and rs13266634 ran in the opposite direction. To assess independence of interactions, we re-estimated the interaction OR for the top findings while adjusting for cis-β-carotene in addition to age, sex, BMI, and race (Figure S8). After adjusting for cis-β-carotene, our inferences were unchanged. For example, for low trans-β-carotene, the per-allele adjusted OR for rs13266634 was 2.0 (95 % CI 1.4–2.8; interaction *p* = 2 × 10^−4^) after adjustment for cis-β-carotene. At high levels of γ-tocopherol, the per-allele OR for rs13266634 was 1.5 (95 % CI 1.1, 1.8; interaction *p* = 0.06). The interaction OR for our top findings was independent of serum cis-β-carotene levels.

### Untransformed environmental factor levels

Environmental factors were log-transformed to achieve linearity. To assess sensitivity of this transformation on our interaction OR, we recomputed models for our top findings without log transforming the environmental factors. We centered and standardized each environmental factor by their raw mean and SD, and recomputed the interaction OR while adjusting for the same covariates above. In conclusion, log transforming the environmental factors did not lead to sizable changes in interaction OR as compared to using the non-logged factors for our top findings (Figure S9). For example, the adjusted OR for participants with 1 SD lower than the mean for trans-β-carotene was 1.8 (95 % CI 1.4–2.5) per one allele change in rs13266634, comparable to the estimate computed using log-transformation of trans-β-carotene.

### Limited evidence to support interactions with other risk factors

BMI, physical fitness, energy intake, carbohydrate intake, and fat intake are well-known risk factors for T2D. We assessed eight variables representing these factors in interaction with the 18 SNPs tested in this study. We were unable to uncover substantial interaction effects that would survive multiple comparison control (Figure S10). The lowest *p* value was 0.02, corresponding to FDR of 100 %. We did observe a modest interaction between BMI and rs8050136 (FTO) (uncorrected interaction *p* value = 0.03). rs8050136 is an obesity-related locus whose association with T2D is explained primarily through its effect on BMI (Zeggini et al. 2007).

## Discussion

We have shown that it is possible to screen for gene–environment interactions by integrating results from GWAS and EWAS. Our most promising results are candidates for prospective studies in additional independent cohorts.

We chose environmental factors and SNPs with strong evidence for marginal associations in EWAS and GWAS. However, it would also be possible to evaluate interactions that lack strong evidence. Given the small marginal effects for most common SNPs, many genuine associations do not reach GWS and remain false negatives (Ioannidis et al. 2011). Some may have strong interactions with environmental factors (Khoury and Wacholder 2009), and may only be discovered if appropriate joint environmental variables are considered. However, choosing them from millions of non-GWS SNPs would be a significant challenge. In addition, testing for interactions is power-intensive (Hunter 2005), and testing a large number would impose a significant power and multiplicity burden (Thomas 2010). It has been argued that strict Bonferroni multiplicity corrections need not be used when considering factors derived from previous observations (Rothman 1990). However, we counter that interaction effects need not exist between factors that have robust evidence from EWAS and GWAS. Further, by estimating the FDR, we present a more powerful way to prioritize findings versus the Bonferroni correction.

Selecting environmental factors to test for interaction is even more difficult. In contrast to SNPs, there is no high-throughput platform that captures environmental factors with low measurement error. This lack of measuring capacity limits data.

We were able to use a prior EWAS to systematically screen 266 T2D-environmental factors measured in serum and urine. We selected five factors with the strongest support for further testing. An advantage of our approach is that it allows for hypothesis generation while keeping the total number of tests lower than testing all possible factor pairs. However, it is still very important to account for multiple hypothesis testing. We used multiplicity correction and FDR, but other approaches may also be employed (Ioannidis 2006). Other alternatives exist to filter the hypothesis space of interactions, such as prioritizing interacting factors based on evidence of physical or toxicological interaction (Patel et al. 2012a).

There were other challenges in this study. First, we had low-to-moderate power to detect moderate interaction effects for some of the interactions we tested. Not surprisingly, the *p* values and effect sizes of results were modest and only one survived Bonferroni correction. We also obtained modest FDR estimates for the other highest-ranking interactions. However, we observed that the top interactions between these SNPs and EWAS factors were stronger than the interactions between the any of the same SNPs and other conventional risk factors for T2D, such as caloric intake, BMI, and physical fitness. We conclude that our top findings are ideal candidates for extensive validation through replication in higher-powered investigations.

Replication studies can investigate trends in SNP interactions with various environmental entities in populations of different ancestry. Population stratification (Smith et al. 2007) is one type of bias for the phenotypic effect of SNPs. Although our analysis adjusted and stratified for race, to date, the SNPs identified by GWAS are best characterized in Caucasian populations. Genetic effects for GWAS-discovered markers may be different in other groups (Hayes et al. 2007; Ioannidis 2009; Shu et al. 2010; Tsai et al. 2010; Unoki et al. 2008; Yamauchi et al. 2010). For example, one study of African–American heart disease patients replicated 17 SNPs found in subjects of European descent. The study identified only one SNP (rs7903146 *TCF7L2*) associated with T2D in African–Americans from a list of 15 SNPs common to this study, including rs13266634 (*SLC30A8*) (Lettre et al. 2011). Little is known about gene–environment interactions in populations of different ancestry and this idea should be investigated.

The potential imbalance of each interaction test was a limitation of this study. Ideally, each interaction pair should have the same participants. However, NHANES subjects did not all undergo the same tests. Our smallest subsamples were those with Heptachlor Epoxide and PCB170. These factors gave high marginal effects, but their analyses were lower powered relative to other subsamples. Our results may be biased and not as generalizable as tests with larger sample sizes.

There are few documented examples of interaction effects between T2D, GWS SNPs and diverse environmental or dietary factors (Cornelis et al. 2009). We have been able to hypothesize about possible new ones. For example, the strongest evidence for interaction in our data was between rs13266634, a non-synonymous coding SNP in the *SLC30A8* gene and three nutrient factors, trans- and cis-β-carotene, and γ-tocopherol. *SLC30A8* is expressed in pancreatic islets and localized in insulin secretory granules of islet β cells. It appears to modulate insulin secretion and storage (Chimienti et al. 2004, 2005). Several reports have found diet-dependent glucose intolerance and insulin secretion abnormalities in *SLC30A8* knockout mice (Lemaire et al. 2009; Nicolson et al. 2009; Pound et al. 2009). rs13266634 has been associated T2D in numerous GWAS [e.g., Sladek et al. (2007), Table S1], and can influence insulin secretion following glucose challenge (Staiger et al. 2007). Thus, this SNP may be important in T2D pathogenesis. Our study enabled us to hypothesize that impaired insulin secretion driven by rs13266634 may increase T2D risk if combined with high or low levels of specific nutrients.

Alternatively, γ-tocopherol and β-carotene may be markers of other dietary components. β-Carotene is a lipid-soluble dietary factor correlated with fruit and vegetable consumption (Block et al. 2001), components that are associated with T2D prevention (Carter et al. 2010). In contrast, the richest sources for γ-tocopherol include soybean oils and margarine (Wagner et al. 2004), components with higher fatty acid content. Fatty acids influence β-cell function and have been shown to even potentiate insulin secretion among individuals genetically predisposed to T2D (Ashcroft and Rorsman 2012). Of interest, vitamin E appears to modify GWAS-identified SNPs associated with serum lipid levels, metabolic traits that are risk factors for T2D (Dumitrescu et al. 2012).

One hypothesis under debate regarding the etiology of T2D is the thrifty genotype hypothesis, in which T2D risk genotypes provided advantages for indigenous human populations. Now, in times of more readily available nutrients and calories, a result of a different environment, these thrifty genotypes are now risk genotypes. However, evidence to support existence of such thrifty genes or interactions with these environmental factors and behaviors is lacking. To this end, competing hypotheses have emerged, including the “thrifty phenotype” (Hales and Barker 2001) and “drifty genotype” (Speakman 2008), whereby predisposition to metabolic diseases are a result of mismatch in nutrition environments between early (pre-childhood) and adult life or due to random genetic drift, respectively. Further, more recent events in human history, such as famine, may have played a role to enrich thrifty genes in certain populations (Diamond 2003). Perhaps one reason behind lack of formal evidence to support these hypotheses may be that other constituents of the modern lifestyle, such as those indicated by EWAS (in addition to higher overall energy intake), may be interacting with genotypes that conferred advantages to early human populations. Future studies should examine the role of other indicators of modern lifestyle and environment on T2D as we have attempted here.

There was some unavoidable asymmetry in our selection of SNPs and environmental factors. We chose SNPs with documented robust associations with T2D and environmental factors with strong associations to T2D in NHANES. Only three variants were significantly associated with T2D overall, and only two were significantly associated with T2D in race-stratified analyses. This pattern was anticipated, given the small marginal effects of these genetic factors.

While interactions may be informative of causality (Davey Smith 2010), these findings are subject to bias. For environmental factors, confounding and reverse causality are major issues (Ioannidis et al. 2009). Little is known about the causal nature, if any, of these factors and T2D (Song et al. 2009). Our findings must be confirmed in independent, larger populations. Prospective studies will be critical.

The SNPs we examined may have robust marginal associations to T2D, but could only tag the actual causal SNP. Our power is decreased for tagging SNPs that are not in complete linkage disequilibrium with the causal SNP. More importantly, etiological inference might be hindered if the causal SNP is unknown.

Nevertheless, these findings may have important implications for personalized medicine (Chan and Ginsburg 2011) or the “missing heritability” debate (Manolio et al. 2009). For example, Roberts et al. (2012) have recently quantified the difficulty in predicting disease risk using entire genomes of individuals. However, Roberts et al. (2012) only considered genetic or environmental main effects and interactions were not considered. On the other hand, Aschard et al. (2012) recently provided theoretical arguments that gene–environment interactions are unlikely to improve risk prediction. However, only a limited number of interactions (maximum of 10) were considered in these simulations. It is possible that inclusion of many interaction effects may increase prediction. We hypothesize that perhaps the lack of predictive capacity in the Roberts et al. investigations and predicted by the Aschard et al. simulations arises from not considering multiple interactions between environmental exposures and the genome. To test the hypothesis empirically that multiple interactions may influence heritability estimates, we would require relatedness information between participants currently unavailable in NHANES. Further, to test if multiple interactions influence risk prediction, we would require samples with same environmental and genetic measures for all participants. Nevertheless, we demonstrate one way of identifying multiple interactions to test in these contexts in future investigations.

Infrastructure-related challenges remain in this area (Hunter 2005). First, unlike common SNPs (Hindorff et al. 2009a), we lack a complete list of candidate environmental factors. Screening and validating gene–environment interactions is power-intensive, and will require both environmental and genetic measures to be measured in multiple studies (Ioannidis et al. 2009), augmentation of GWAS with environmental data (Khoury and Wacholder 2009), and adoption of measurement standards (e.g., Hamilton et al. 2011). A systematic approach to investigating the interactions of environment and the individual genome may help explain a substantial component of disease risk, lead to hypotheses regarding disease pathology, or help shed light on the debate on the genetic basis of disease (Gibson 2011).

## Electronic supplementary material

Below is the link to the electronic supplementary material.

Supplementary material 1 (PDF 6289 kb)

## Abbreviations

T2D: Type 2 diabetes mellitus
GWAS: Genome-wide association study
EWAS: Environment-wide association study
GWS: Genome-wide significance
NHANES: National Health and Nutrition Examination Survey
FDR: False discovery rate
OR: Odds ratio
SNP: Single nucleotide polymorphism
BMI: Body mass index
FBG: Fasting blood glucose
T1D: Type 1 diabetes
CEU: HapMap population of European ancestry living in Utah
MEX: HapMap population of Mexican ancestry from Los Angeles
ASW: HapMap population of African ancestry from Southwestern United States
NCHS: National Centers for Health Statistics
RDC: Research Data Center
HWE: Hardy–Weinberg equilibrium
