# Supplementary material for: Systematic identification of interaction effects between genome- and environment-wide associations in type 2 diabetes mellitus
Source: Hum Genet. 2013 Jan 20;132(5):495–508. doi: 10.1007/s00439-012-1258-z (PMC3625410; doi:10.1007/s00439-012-1258-z)
Supplement: Supplementary file 1 — Supplementary material 1 (PDF 6289 kb) [file 439_2012_1258_MOESM1_ESM.pdf]

Systematic identification of interaction effects between genome- and environment-wide associations in Type 2 Diabetes Mellitus

Chirag J. Patel, Rong Chen,Keiichi Kodama, John P. A. Ioannidis, Atul J. Butte

Supplementary Materials

Table S1. Examples of genome-wide association studies and publications for 18 selected SNPs.

Table S2. Correlation matrix (Pearson's ρ) of environmental factors

Figure S1. Allele specification, risk allele frequency, and marginal effect size (adjusted odds ratio) for 18 loci.

Figure S2. Serum levels of environmental factors and marginal effect sizes (adjusted odds ratio) for five factors.

Figure S3. Sample sizes and power for detection of interaction per SNP by environmental factor pair.

Figure S4. Risk allele frequency and genetic marginal effect sizes per subsample with joint measures for genetic variation and environmental factors.

Figure S5. Correlation of SNP by environmental factor.

Figure S6. Per-SD environmental factor OR for top interaction.

Figure S7. Per-risk allele effect sizes for Mexican-Americans and Non-Hispanic Blacks.

Figure S8. Per-risk allele effect sizes adjusting for cis-β-carotene.

Figure S9. Per-risk allele effect sizes at non-logged levels of environmental factors.

Figure S10. –log10(p-values) for interaction between SNPs and other factors.

References

rsid (gene)	Population of sample(s)	OR/association p-value(s)	reference(s)
Rs10923931 (NOTCH2)	Caucasian (European)	1.13/1x10-8	(Zeggini et al. 2008)
Rs7903146 (TCF7L2)	Caucasian (European), Caucasian (American; European), Japanese, Indian	1.65(het)/3x10-10 , 1.4/1x10-48, 1.5/8x10-12 ,7x10-7	(Saxena et al. 2007; Sladek et al. 2007; Takeuchi et al. 2009; Zabaneh and Balding 2010)
Rs13266634 (SLC30A8)	Caucasian, Caucasian (American; European), Japanese	1.18(het)/6x10-8, 1.12/5x10-8, 1.2/2x10-14	(Saxena et al. 2007; Sladek et al. 2007; Takeuchi et al. 2009)
Rs7901695 (TCF7L2)	Caucasian (European), African American	1.37/1x10-48, 1.2/1x10-6	(Lettre et al. 2011; Zeggini et al. 2007)
Rs2383208 (CDKN2A)	Japanese	1.3/2x10-29	(Takeuchi et al. 2009)
Rs1260326 (GCKR)	European	0.07 (2-hour glucose)/7x10-11	(Saxena et al. 2010) (glucose challenge)
Rs780094 (GCKR)	European	1x10-9	(Dupuis et al. 2010)
Rs2237895 (KCNQ1)	Japanese, Chinese	1.4/2x10-42,1.29/1x10-9	(Yasuda et al. 2008),(Tsai et al. 2010)
Rs10811661 (unknown)	Caucasian (European), Caucasian (European; American), Korean	1.19/5x10-8, 1.2/8x10-15, 1.55/2x10-8	(Ng et al. 2008; Saxena et al. 2007; Scott et al. 2007)
Rs4712523 (CDKAL1)	Caucasian (European), Japanese	1.2/2x10-12, 1.27/7x10-20	(Rung et al. 2009; Takeuchi et al. 2009)
Rs4607103 (ADAMTS9)	Caucasian (European)	1.09/1x10-9	(Zeggini et al. 2008)
Rs1111875 (HHEX)	Caucasian, Japanese	1.13/6x10-10, 1.21/7x10-12	(Saxena et al. 2007; Takeuchi et al. 2009)
Rs7578597 (THADA)	Caucasian (European)	1.15/1x10-9	(Zeggini et al. 2008)
Rs4402960 (IGFBP2)	Caucasian (American; European)	1.17/1.7x10-9	(Saxena et al. 2007)
Rs1801282 (PPARG)	Caucasian (American;European), Caucasian	1.1/5x10-8,1.14/8x10-6	(Saxena et al. 2007; Voight et al. 2010)
Rs12779790 (CAMK1D)	Caucasian	1.11/1x10-10	(Zeggini et al. 2008)

Rs8050136 (FTO)	Caucasian (European), Caucasian (European)	1.23/9x10-16, 1.17/1x10-12	(Scott et al. 2007; Zeggini et al. 2007)
Rs864745 (JAZF1)	Caucasian (European), Caucasian	1.10/5x10-14, 1.12x10-8	(Voight et al. 2010; Zeggini et al. 2008)

Table S1. Examples of genome-wide association studies and publications for 18 selected SNPs.

	Heptachlor Epoxide	PCB170	g-tocopherol	trans-b-carotene	cis-b-carotene
Heptachlor Epoxide	1	0.34	0.13	0.04	0.04
PCB170	0.34	1	0.03	0.19	0.2
g-tocopherol	0.13	0.03	1	-0.22	-0.2
trans-b-carotene	0.04	0.19	-0.22	1	0.96
cis-b-carotene	0.04	0.2	-0.2	0.96	1

Table S2. Correlation matrix (Pearson's ρ) of environmental factors.

rsid(gene)	major/minor/risk	race	risk %	N(cases)	OR (95% CI)	p
rs10923931(NOTCH2)	G/T/T	all	0.12	3138(291)	1.51(1.14,1.99)	0.004
		W	0.1	1624(124)	1.39(0.96,2.01)	0.082
		M	0.09	778(91)	1.43(0.75,2.73)	0.272
		B	0.31	494(51)	1.17(0.82,1.66)	0.389
		OH	0.14	154(16)		
		O	0.11	88(9)		
rs7903146(TCF7L2)	C/T/T	all	0.28	3111(290)	1.32(1.06,1.65)	0.015
		W	0.29	1617(121)	1.43(1.06,1.93)	0.019
		M	0.21	768(91)	1.15(0.88,1.51)	0.313
		B	0.31	489(53)	1.09(0.75,1.6)	0.647
		OH	0.3	151(16)		
		O	0.15	86(9)		
rs7901695(TCF7L2)	T/C/C	all	0.31	3115(287)	1.21(0.94,1.57)	0.14
		W	0.31	1612(122)	1.33(0.95,1.85)	0.096
		M	0.23	773(89)	1.03(0.78,1.34)	0.852
		B	0.46	495(51)	0.97(0.61,1.57)	0.914
		OH	0.34	150(16)		
		O	0.16	85(9)		
rs13266634(SLC30A8)	C/T/C	all	0.72	3135(292)	1.32(1.04,1.67)	0.022
		W	0.7	1624(124)	1.37(1,1.87)	0.051
		M	0.73	778(91)	1.29(0.77,2.16)	0.341
		B	0.91	491(52)	1.17(0.47,2.92)	0.73
		OH	0.75	154(16)		
		O	0.7	88(9)		
rs2383208(intergenic)	A/G/A	all	0.81	3130(292)	0.88(0.6,1.27)	0.482
		W	0.81	1621(124)	1(0.59,1.71)	0.996
		M	0.86	777(91)	0.98(0.69,1.39)	0.914
		B	0.81	492(52)	0.66(0.32,1.37)	0.268
		OH	0.81	152(16)		
		O	0.71	88(9)		
rs1260326(GCKR)	C/T/C	all	0.63	3118(290)	1.27(0.93,1.73)	0.126
		W	0.6	1617(121)	1.43(0.94,2.2)	0.098
		M	0.67	772(91)	1.04(0.71,1.53)	0.835
		B	0.85	490(53)	1.21(0.8,1.83)	0.37
		OH	0.64	151(16)		
		O	0.63	88(9)		
rs780094(GCKR)	G/A/G	all	0.63	3138(292)	1.25(0.92,1.7)	0.153
		W	0.61	1622(124)	1.39(0.93,2.08)	0.11
		M	0.67	783(91)	1(0.65,1.54)	0.993
		B	0.82	492(52)	1.38(0.9,2.12)	0.141
		OH	0.62	152(16)		
		O	0.65	89(9)		
rs2237895(KCNQ1)	A/C/C	all	0.39	3125(290)	0.95(0.78,1.17)	0.657
		W	0.42	1613(122)	0.97(0.72,1.33)	0.867
		M	0.44	781(91)	0.97(0.64,1.46)	0.878
		B	0.17	493(52)	1.67(1.02,2.75)	0.043
		OH	0.37	150(16)		
		O	0.34	88(9)		
rs10811661(intergenic)	T/C/T	all	0.83	3116(290)	0.97(0.62,1.5)	0.886
		W	0.82	1616(121)	1.02(0.58,1.79)	0.945
		M	0.87	772(91)	1.07(0.71,1.62)	0.748
		B	0.94	490(53)	1.69(0.68,4.2)	0.259
		OH	0.84	151(16)		
		O	0.7	87(9)		

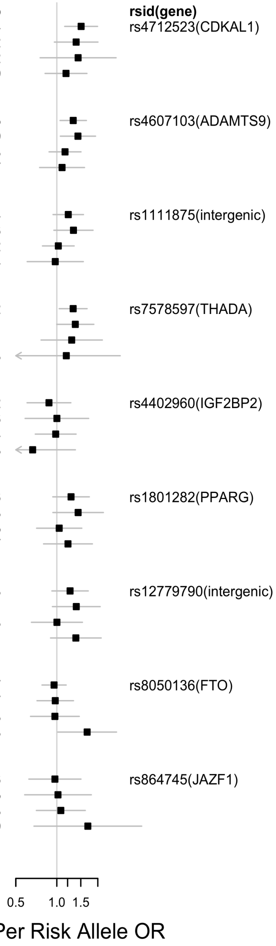

rsid(gene)	major/minor/risk	race	risk %	N(cases)	OR (95% CI)	p
rs4712523(CDKAL1)	A/G/G	all	0.34	3139(292)	1.07(0.81,1.42)	0.627
		W	0.31	1622(124)	1.16(0.79,1.72)	0.445
		M	0.3	783(91)	1.26(0.88,1.8)	0.213
		B	0.6	493(52)	0.68(0.44,1.03)	0.07
		OH	0.35	152(16)		
		O	0.37	89(9)		
rs4607103(ADAMTS9)	C/T/C	all	0.73	3129(292)	0.89(0.68,1.18)	0.418
		W	0.75	1622(124)	0.82(0.58,1.15)	0.25
		M	0.68	777(91)	1.02(0.6,1.73)	0.94
		B	0.7	490(52)	0.69(0.48,1.01)	0.058
		OH	0.66	152(16)		
		O	0.67	88(9)		
rs1111875(intergenic)	G/A/G	all	0.6	3117(290)	0.97(0.78,1.21)	0.792
		W	0.59	1617(121)	0.93(0.72,1.2)	0.569
		M	0.63	771(91)	0.88(0.54,1.44)	0.609
		B	0.75	490(53)	1.07(0.61,1.89)	0.818
		OH	0.61	151(16)		
		O	0.48	88(9)		
rs7578597(THADA)	T/C/T	all	0.89	3130(286)	1.13(0.76,1.68)	0.538
		W	0.89	1623(123)	1.19(0.72,1.99)	0.496
		M	0.94	775(89)	1.72(1.04,2.85)	0.034
		B	0.76	496(50)	1.15(0.69,1.9)	0.601
		OH	0.91	149(15)		
		O	0.93	87(9)		
rs4402960(IGF2BP2)	G/T/T	all	0.33	3116(290)	0.92(0.73,1.16)	0.498
		W	0.32	1616(121)	0.99(0.74,1.33)	0.946
		M	0.25	772(91)	1.09(0.74,1.63)	0.659
		B	0.52	490(53)	0.85(0.52,1.4)	0.521
		OH	0.32	151(16)		
		O	0.28	87(9)		
rs1801282(PPARG)	C/G/C	all	0.89	3115(290)	0.89(0.58,1.36)	0.583
		W	0.88	1617(121)	0.82(0.5,1.34)	0.428
		M	0.88	771(91)	1.28(0.67,2.45)	0.459
		B	0.97	489(53)	0.55(0.21,1.44)	0.223
		OH	0.9	151(16)		
		O	0.94	87(9)		
rs12779790(intergenic)	A/G/G	all	0.18	3127(288)	0.92(0.66,1.28)	0.605
		W	0.18	1621(123)	0.96(0.64,1.45)	0.862
		M	0.18	776(89)	0.62(0.39,0.99)	0.045
		B	0.16	493(52)	1.06(0.61,1.85)	0.832
		OH	0.16	149(15)		
		O	0.18	88(9)		
rs8050136(FTO)	C/A/A	all	0.38	3114(289)	0.96(0.75,1.24)	0.752
		W	0.39	1618(120)	0.88(0.62,1.26)	0.494
		M	0.24	770(91)	1.68(1.22,2.3)	0.001
		B	0.44	489(53)	1.33(0.97,1.84)	0.077
		OH	0.33	150(16)		
		O	0.26	87(9)		
rs864745(JAZF1)	A/G/A	all	0.55	3138(292)	0.94(0.69,1.29)	0.722
		W	0.51	1621(124)	0.93(0.64,1.36)	0.711
		M	0.62	784(91)	0.93(0.59,1.47)	0.77
		B	0.76	492(52)	1.55(0.88,2.73)	0.132
		OH	0.6	152(16)		
		O	0.68	89(9)		

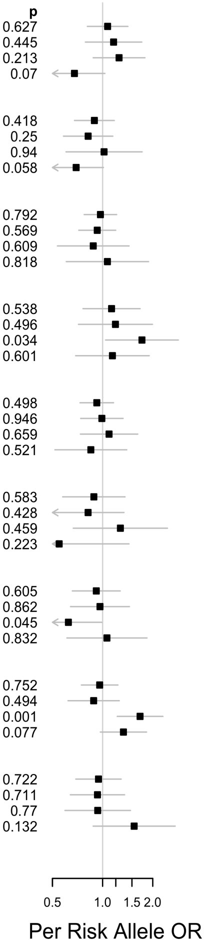

Figure S1. Allele specification, risk allele frequency, and marginal effect size (adjusted odds ratio) for 18 loci. Race key: "All": All races combined; "W": Non-Hispanic White; "M": Mexican-American; "B": Non-Hispanic Black. All effect sizes were adjusted by age, sex, BMI. The "All" model was adjusted by age, sex, BMI, and race. Risk allele specification determined from the literature.

factor	race	N(cases)	Diabetics Mean (95% CI) [units]	Controls Mean (95% CI) [units]	OR (95% CI)	p
PCB170	all	1710(97)	0.14(0.12,0.16) [ng/g]	0.08(0.07,0.08) [ng/g]	1.72(1.18,2.51)	0.011
	W	728(38)	0.14(0.11,0.16) [ng/g]	0.08(0.08,0.09) [ng/g]	1.59(1.04,2.43)	0.045
	M	491(28)	0.12(0.07,0.18) [ng/g]	0.05(0.04,0.05) [ng/g]	1.85(0.81,4.25)	0.172
	B	358(20)	0.17(0.11,0.23) [ng/g]	0.07(0.06,0.08) [ng/g]	2.15(0.72,6.41)	0.191
	OH	74(5)	0.1(0.05,0.15) [ng/g]	0.06(0.04,0.08) [ng/g]		
	O	59(6)	0.15(0.11,0.19) [ng/g]	0.06(0.04,0.07) [ng/g]		
trans-b-carotene	all	2847(186)	13.32(10.82,15.82) [ug/dL]	17.23(16.22,18.24) [ug/dL]	0.63(0.51,0.78)	0.003
	W	1320(82)	14.28(10.65,17.91) [ug/dL]	17.75(16.29,19.22) [ug/dL]	0.68(0.52,0.91)	0.022
	M	722(50)	15.69(12.24,19.13) [ug/dL]	16.9(14.83,18.97) [ug/dL]	0.76(0.55,1.04)	0.16
	B	601(36)	10.2(7.29,13.1) [ug/dL]	15.34(13.23,17.44) [ug/dL]	0.52(0.38,0.73)	0.005
	OH	99(9)	15.05(7.34,22.76) [ug/dL]	13.47(10.44,16.5) [ug/dL]		
	O	105(9)	7.68(2.79,12.57) [ug/dL]	17.79(14.16,21.42) [ug/dL]		
cis-b-carotene	all	2846(186)	0.82(0.7,0.94) [ug/dL]	1(0.95,1.05) [ug/dL]	0.62(0.48,0.79)	0.006
	W	1319(82)	0.85(0.67,1.03) [ug/dL]	1.02(0.95,1.09) [ug/dL]	0.67(0.49,0.9)	0.022
	M	722(50)	0.91(0.72,1.1) [ug/dL]	0.93(0.83,1.04) [ug/dL]	0.8(0.58,1.09)	0.231
	B	601(36)	0.71(0.56,0.86) [ug/dL]	0.99(0.86,1.11) [ug/dL]	0.47(0.26,0.83)	0.032
	OH	99(9)	0.92(0.62,1.22) [ug/dL]	0.77(0.62,0.92) [ug/dL]		
	O	105(9)	0.6(0.35,0.85) [ug/dL]	1.05(0.85,1.26) [ug/dL]		
g-tocopherol	all	5039(310)	327.94(295.49,360.39) [ug/dL]	225.72(216.78,234.65) [ug/dL]	1.47(1.25,1.73)	<0.001
	W	2193(131)	324.86(276.47,373.26) [ug/dL]	225.92(214.48,237.36) [ug/dL]	1.35(1.15,1.59)	0.001
	M	1343(84)	376.45(300.05,452.85) [ug/dL]	223.38(211.34,235.41) [ug/dL]	1.78(1.33,2.39)	0.002
	B	1087(64)	359.6(316.47,402.74) [ug/dL]	241.76(233.42,250.1) [ug/dL]	3.2(1.66,6.19)	0.002
	OH	241(18)	249.78(200.64,298.93) [ug/dL]	199.78(180.77,218.8) [ug/dL]		
	O	175(13)	343(194.47,491.53) [ug/dL]	225.95(201.01,250.88) [ug/dL]		
Heptachlor Epoxide	all	1618(93)	0.13(0.09,0.18) [ng/g]	0.05(0.05,0.06) [ng/g]	1.49(1.13,1.98)	0.011
	W	683(35)	0.14(0.07,0.21) [ng/g]	0.05(0.05,0.06) [ng/g]	1.66(1.17,2.35)	0.009
	M	488(29)	0.26(0,0.52) [ng/g]	0.05(0.04,0.06) [ng/g]	1.73(0.92,3.26)	0.122
	B	331(19)	0.08(0.06,0.11) [ng/g]	0.04(0.03,0.04) [ng/g]	1.61(0.84,3.07)	0.173
	OH	63(5)	0.12(-0.01,0.25) [ng/g]	0.04(0.03,0.05) [ng/g]		
	O	53(5)	0.04(0.02,0.05) [ng/g]	0.05(0.03,0.06) [ng/g]		

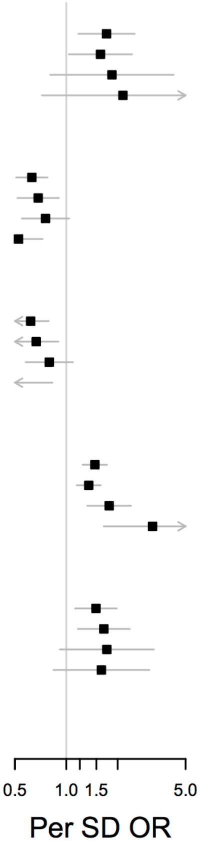

Figure S2. Serum levels of environmental factors and marginal effect sizes (adjusted odds ratio) for five factors. Race key: "All": all races combined; "W": Non-Hispanic White; "M": Mexican American; "B": Non-Hispanic Black, "OH": Other Hispanic; "O": Other.. Means are specified in their original units of measure (ng/g: nanogram per gram serum, ug/mL: microgram per milliliter). Adjusted odds ratios were adjusted by age, sex, and BMI; The "All" model was adjusted by age, sex, BMI, and race. Adjusted odds ratios are for a change in 1SD of logged exposure value.

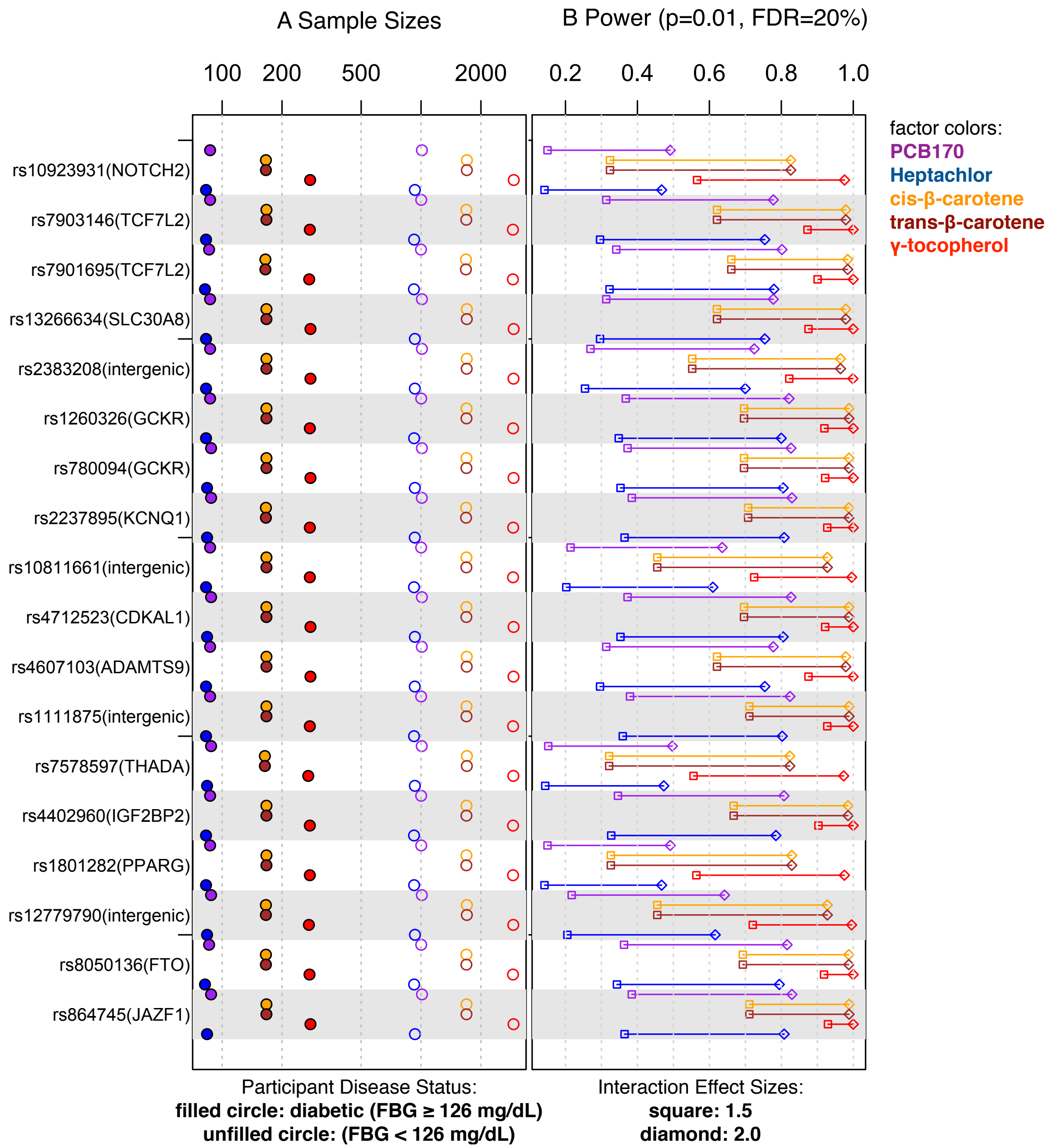

Figure S3. Sample sizes and power for detection of interaction per SNP by environmental factor pair. A) Sample size for each SNP and environmental factor pair. Filled markers correspond to diabetics. B) Power estimate for detection of interaction for each genetic locus and environmental factor pair tested against T2D. Assumptions include an interaction odds ratio of 1.5 and 2.0, a main effect of the SNP of 1.0 (additive model) and environmental factor OR of 1.5, the minor allele frequencies as in Figure 2 (combined races), 10 controls per case, environmental factor SD of 1, and p-value of 0.01 (FDR of 18%). FBG: fasting blood glucose.

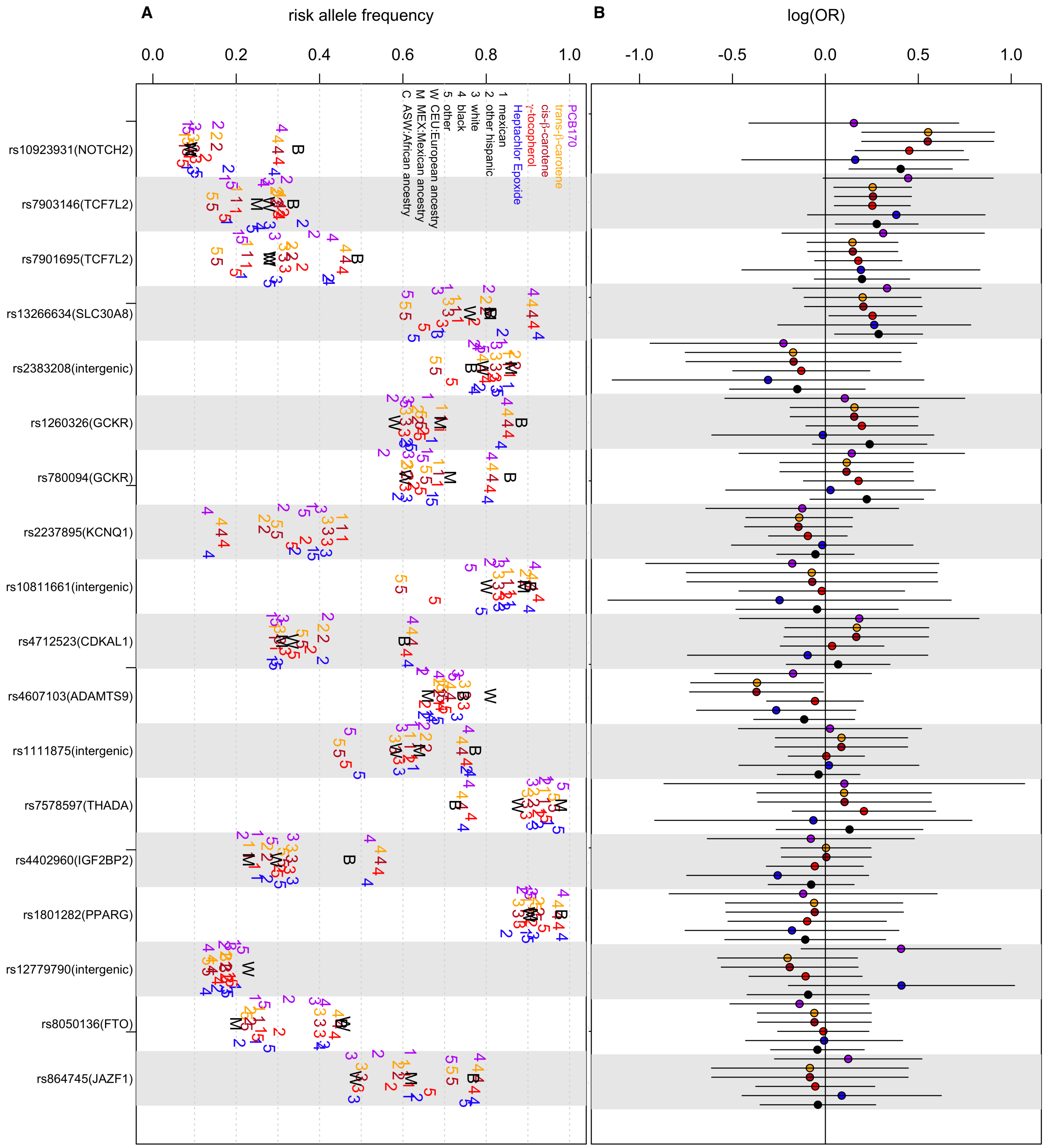

Figure S4. Risk allele frequency and genetic marginal effect sizes per subsample with joint measures for genetic variation and environmental factors. A) Risk allele frequency by race. Numbers indicate ancestry (1: Mexican American; 2: Other Hispanic; 3: Non-Hispanic White; 4: Non-Hispanic Black; 5: Other); letter indicates minor allele frequency in HapMap populations (W: CEU, European; M: MEX, Mexican; B: ASW, African). 2) Marginal effect size (log(odds ratio)) adjusted by sex, age, race, BMI. The black marker denotes the overall genetic effect size in the full sample. Colored markers indicate environmental factors: red: γ-tocopherol, blue: heptachlor epoxide, purple: PCB170, brown: cis-β-carotene, orange: trans-β-carotene.

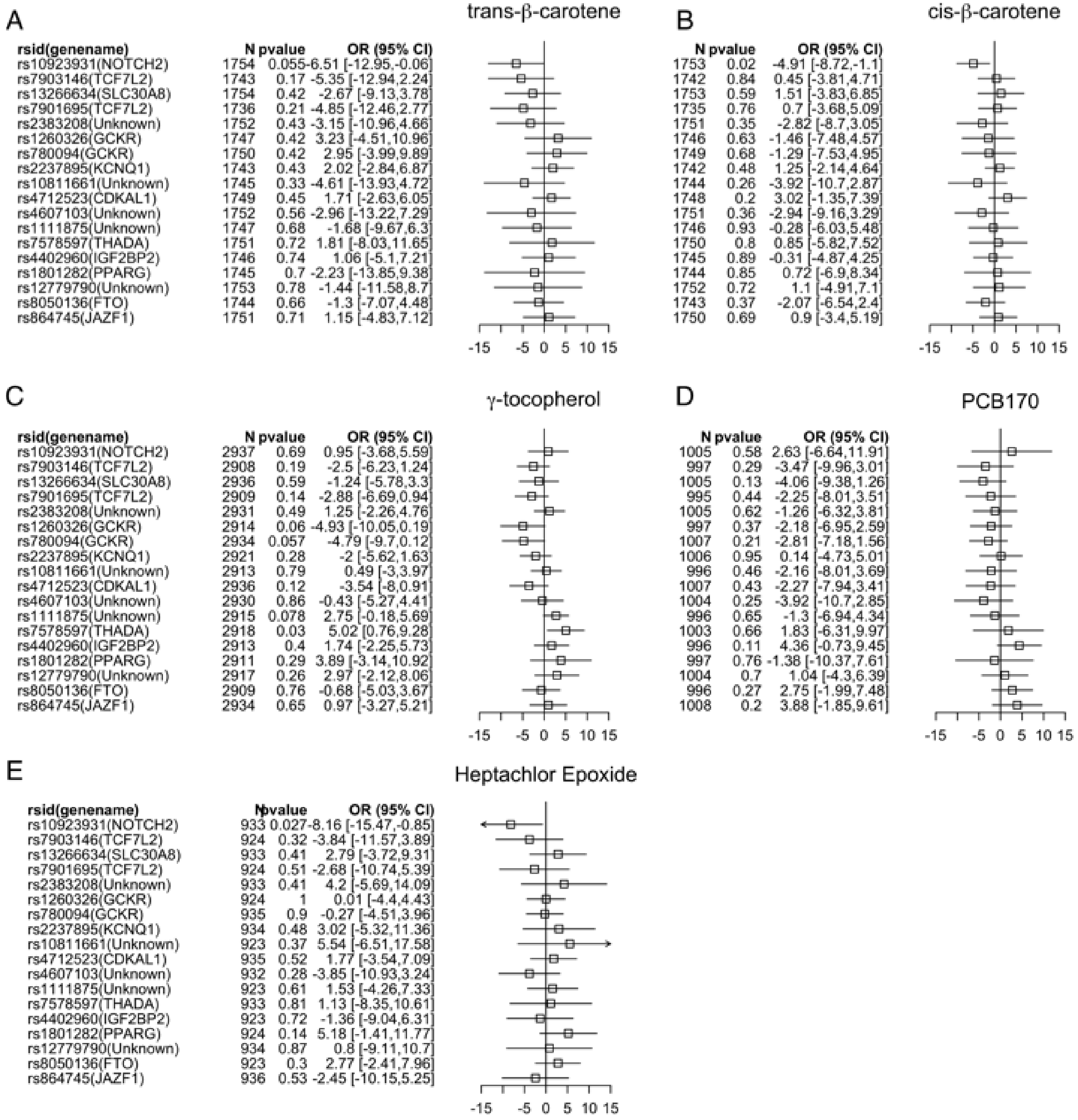

Figure S5. Correlation of SNP by environmental factor. Each locus was evaluated for differences in levels of A) PCB170, B) heptachlor epoxide, C) γ-tocopherol, D) cis-β-carotene, and E) trans-β-carotene, depending on the number of risk alleles at each locus, while adjusting for sex, age, self-report race, and BMI. X-axis: units of percent change of exposure for 1 risk allele. All p-values are uncorrected for multiple hypotheses.

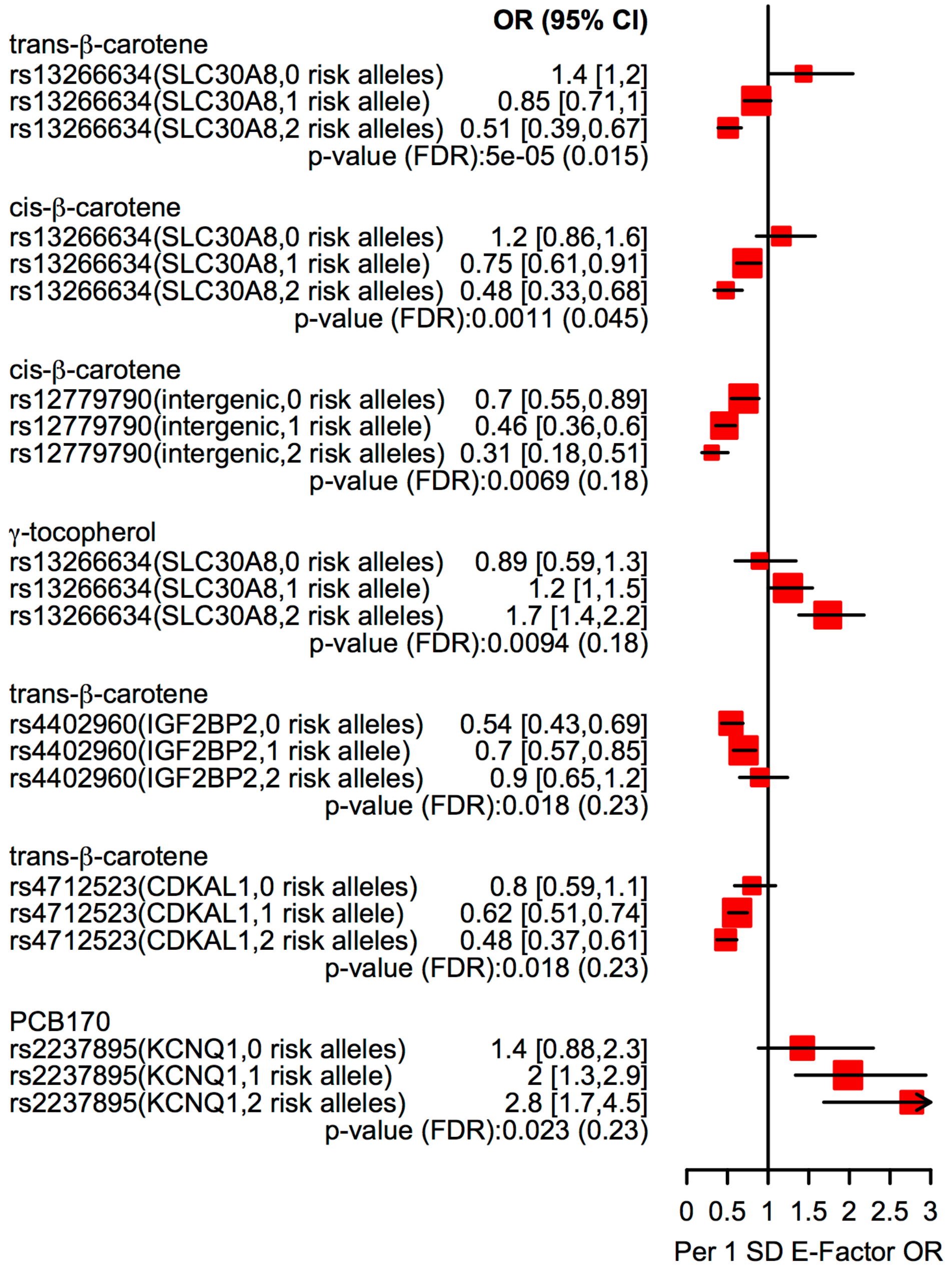

Figure S6. Per-SD environmental factor OR for top interactions. Red markers indicate effects computed conditional on 0, 1, or 2 risk alleles respectively. Marker sizes are proportional to inverse variance.

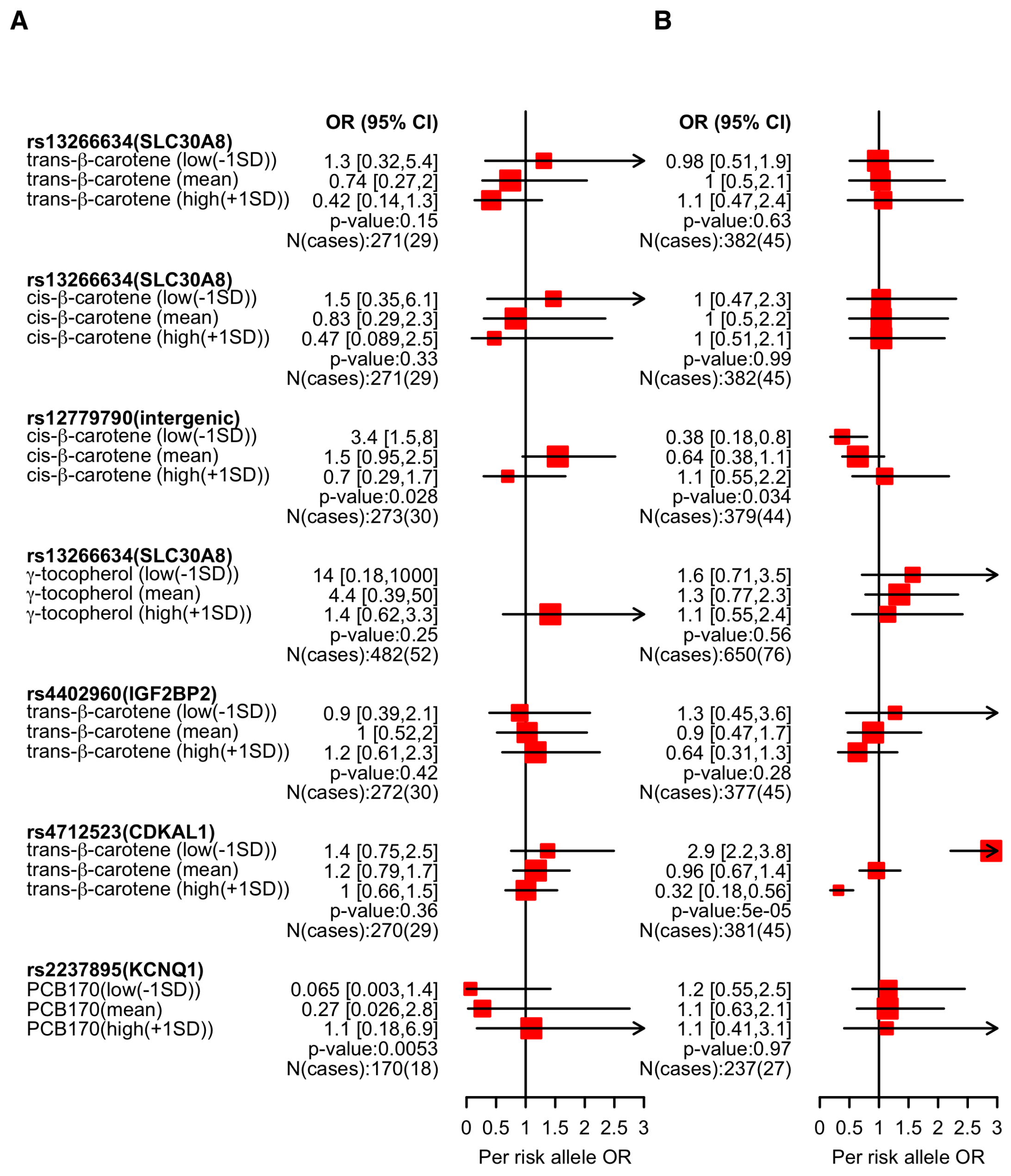

Figure S7. Per-risk allele effect sizes for Mexican-Americans and Non-Hispanic Blacks. (**A**) Non-Hispanic Blacks, (**B**) Mexican Americans. Markers denote interaction OR computed at 1SD below mean exposure levels, at the mean, or at 1SD above the mean. Marker sizes are proportional to inverse variance.

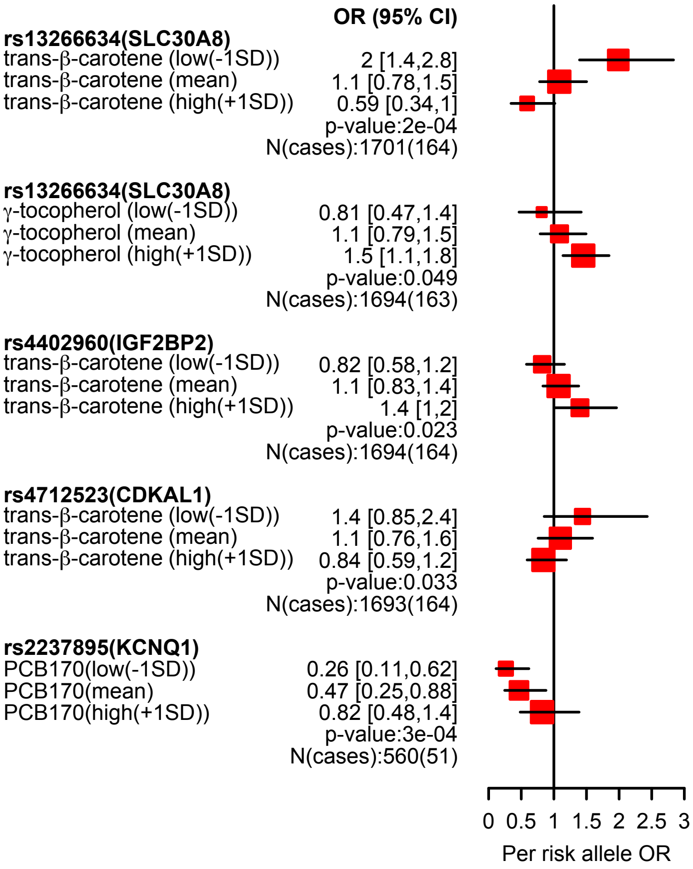

Figure S8. Per-risk allele effect sizes adjusting for cis-β-carotene. Markers denote interaction OR computed at 1SD below mean exposure levels, at the mean, or at 1SD greater than the mean. Marker sizes are proportional to inverse variance. Models were adjusted by age, sex, race, BMI, and cis-β-carotene.

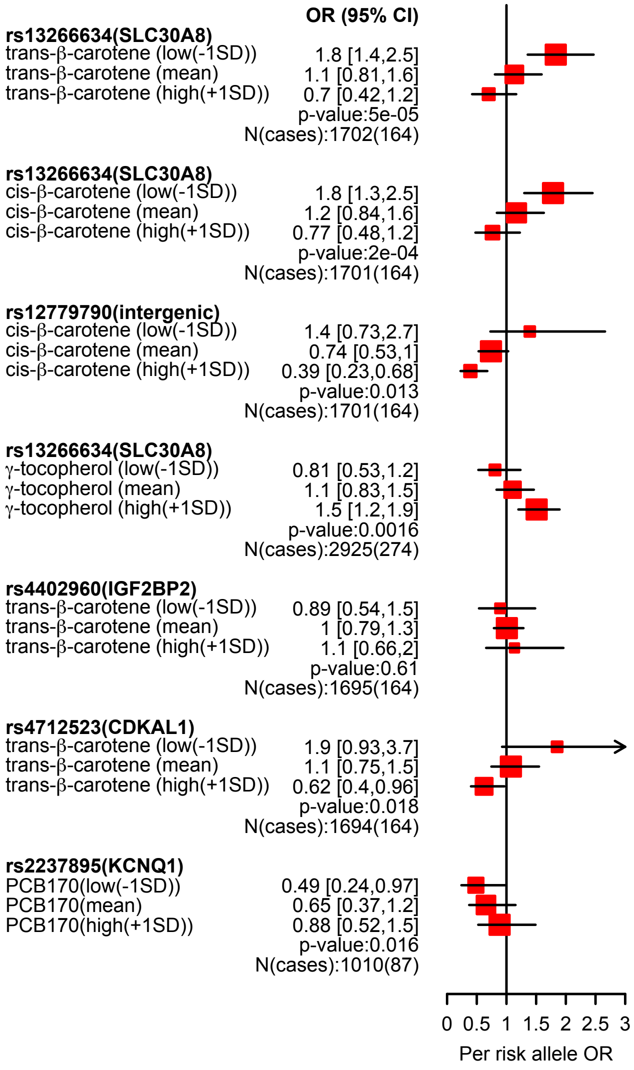

Figure S9. Per-risk allele effect sizes at non-logged levels of environmental factors. Markers denote interaction OR computed at 1SD below mean exposure levels, at the mean, or at 1SD greater than the mean. Marker sizes are proportional to inverse variance.

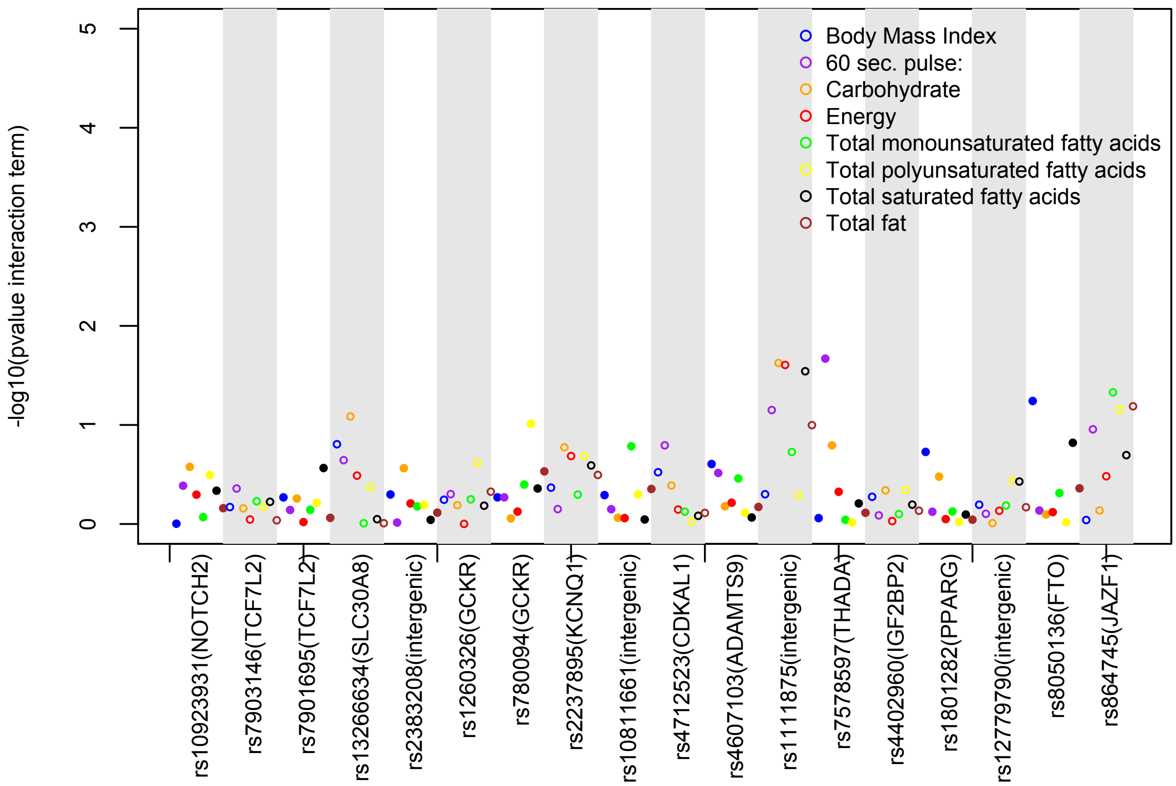

Figure S10. –log10(p-values) for interaction between SNPs and other factors. Blue: Body Mass Index, Purple: 60 second pulse rate, Orange: Total 24-hour carbohydrate intake, Red: Total 24-hour energy intake, Green: Total 24-hour monounsaturated fat intake, Yellow: Total 24-hour polyunsaturated fat intake, Black: Total 24-hour saturated fat intake, Brown: Total 24-hour fat intake.

References

Dupuis J, Langenberg C, Prokopenko I, Saxena R, Soranzo N, Jackson AU, Wheeler E, Glazer NL, Bouatia-Naji N, Gloyn AL, Lindgren CM, Magi R, Morris AP, Randall J, Johnson T, Elliott P, Rybin D, Thorleifsson G, Steinthorsdottir V, Henneman P, Grallert H, Dehghan A, Hottenga JJ, Franklin CS, Navarro P, Song K, Goel A, Perry JR, Egan JM, Lajunen T, Grarup N, Sparso T, Doney A, Voight BF, Stringham HM, Li M, Kanoni S, Shrader P, Cavalcanti-Proenca C, Kumari M, Qi L, Timpson NJ, Gieger C, Zabena C, Rocheleau G, Ingelsson E, An P, O'Connell J, Luan J, Elliott A, McCarroll SA, Payne F, Roccasecca RM, Pattou F, Sethupathy P, Ardlie K, Ariyurek Y, Balkau B, Barter P, Beilby JP, Ben-Shlomo Y, Benediktsson R, Bennett AJ, Bergmann S, Bochud M, Boerwinkle E, Bonnefond A, Bonnycastle LL, Borch-Johnsen K, Bottcher Y, Brunner E, Bumpstead SJ, Charpentier G, Chen YD, Chines P, Clarke R, Coin LJ, Cooper MN, Cornelis M, Crawford G, Crisponi L, Day IN, de Geus EJ, Delplanque J, Dina C, Erdos MR, Fedson AC, Fischer-Rosinsky A, Forouhi NG, Fox CS, Frants R, Franzosi MG, Galan P, Goodarzi MO, Graessler J, Groves CJ, Grundy S, Gwilliam R, Gyllensten U, Hadjadj S, et al. (2010) New genetic loci implicated in fasting glucose homeostasis and their impact on type 2 diabetes risk. Nat Genet 42: 105-16. doi: 10.1038/ng.520

Lettre G, Palmer CD, Young T, Ejebe KG, Allayee H, Benjamin EJ, Bennett F, Bowden DW, Chakravarti A, Dreisbach A, Farlow DN, Folsom AR, Fornage M, Forrester T, Fox E, Haiman CA, Hartiala J, Harris TB, Hazen SL, Heckbert SR, Henderson BE, Hirschhorn JN, Keating BJ, Kritchevsky SB, Larkin E, Li M, Rudock ME, McKenzie CA, Meigs JB, Meng YA, Mosley TH, Newman AB, Newton-Cheh CH, Paltoo DN, Papanicolaou GJ, Patterson N, Post WS, Psaty BM, Qasim AN, Qu L, Rader DJ, Redline S, Reilly MP, Reiner AP, Rich SS, Rotter JI, Liu Y, Shrader P, Siscovick DS, Tang WH, Taylor HA, Tracy RP, Vasan RS, Waters KM, Wilks R, Wilson JG, Fabsitz RR, Gabriel SB, Kathiresan S, Boerwinkle E (2011) Genome-wide association study of coronary heart disease and its risk factors in 8,090 African Americans: the NHLBI CARe Project. PLoS Genet 7: e1001300. doi: 10.1371/journal.pgen.1001300

Ng MC, Park KS, Oh B, Tam CH, Cho YM, Shin HD, Lam VK, Ma RC, So WY, Cho YS, Kim HL, Lee HK, Chan JC, Cho NH (2008) Implication of genetic variants near TCF7L2, SLC30A8, HHEX, CDKAL1, CDKN2A/B, IGF2BP2, and FTO in type 2 diabetes and obesity in 6,719 Asians. Diabetes 57: 2226-33. doi: 10.2337/db07-1583

Rung J, Cauchi S, Albrechtsen A, Shen L, Rocheleau G, Cavalcanti-Proenca C, Bacot F, Balkau B, Belisle A, Borch-Johnsen K, Charpentier G, Dina C, Durand E, Elliott P, Hadjadj S, Jarvelin MR, Laitinen J, Lauritzen T, Marre M, Mazur A, Meyre D, Montpetit A, Pisinger C, Posner B, Poulsen P, Pouta A, Prentki M, Ribel-Madsen R, Ruokonen A, Sandbaek A, Serre D, Tichet J, Vaxillaire M, Wojtaszewski JF, Vaag A, Hansen T, Polychronakos C, Pedersen O, Froguel P, Sladek R (2009) Genetic variant near IRS1 is associated with type 2 diabetes, insulin resistance and hyperinsulinemia. Nat Genet 41: 1110-5. doi: 10.1038/ng.443

Saxena R, Hivert MF, Langenberg C, Tanaka T, Pankow JS, Vollenweider P, Lyssenko V, Bouatia-Naji N, Dupuis J, Jackson AU, Kao WH, Li M, Glazer NL, Manning AK, Luan J, Stringham HM, Prokopenko I, Johnson T, Grarup N, Boesgaard TW, Lecoeur C, Shrader P, O'Connell J, Ingelsson E, Couper DJ, Rice K, Song K, Andreasen CH, Dina C, Kottgen A, Le Bacquer O, Pattou F, Taneera J, Steinthorsdottir V, Rybin D, Ardlie K, Sampson M, Qi L, van Hoek M, Weedon MN, Aulchenko YS, Voight BF, Grallert H, Balkau B, Bergman RN, Bielinski SJ, Bonnefond A, Bonnycastle LL, Borch-Johnsen K, Bottcher Y, Brunner E, Buchanan TA, Bumpstead SJ, Cavalcanti-Proenca C, Charpentier G, Chen YD, Chines PS, Collins FS, Cornelis M, G JC, Delplanque J, Doney A, Egan JM, Erdos MR, Firmann M, Forouhi NG, Fox CS, Goodarzi MO, Graessler J, Hingorani A, Isomaa B, Jorgensen T, Kivimaki M, Kovacs P, Krohn K, Kumari M, Lauritzen T, Levy-Marchal C, Mayor V, McAteer JB, Meyre D, Mitchell BD, Mohlke KL, Morken MA, Narisu N, Palmer CN, Pakyz R, Pascoe L, Payne F, Pearson D, Rathmann W, Sandbaek A, Sayer AA, Scott LJ, Sharp SJ, Sijbrands E, Singleton A, Siscovick DS, Smith NL, Sparso T, et al. (2010) Genetic variation in GIPR influences the glucose and insulin responses to an oral glucose challenge. Nat Genet 42: 142-8. doi: 10.1038/ng.521

Saxena R, Voight BF, Lyssenko V, Burtt NP, de Bakker PI, Chen H, Roix JJ, Kathiresan S, Hirschhorn JN, Daly MJ, Hughes TE, Groop L, Altshuler D, Almgren P, Florez JC, Meyer J, Ardlie K, Bengtsson

Bostrom K, Isomaa B, Lettre G, Lindblad U, Lyon HN, Melander O, Newton-Cheh C, Nilsson P, Orho-Melander M, Rastam L, Speliotes EK, Taskinen MR, Tuomi T, Guiducci C, Berglund A, Carlson J, Gianniny L, Hackett R, Hall L, Holmkvist J, Laurila E, Sjogren M, Sterner M, Surti A, Svensson M, Tewhey R, Blumenstiel B, Parkin M, Defelice M, Barry R, Brodeur W, Camarata J, Chia N, Fava M, Gibbons J, Handsaker B, Healy C, Nguyen K, Gates C, Sougnez C, Gage D, Nizzari M, Gabriel SB, Chirn GW, Ma Q, Parikh H, Richardson D, Ricke D, Purcell S (2007) Genome-wide association analysis identifies loci for type 2 diabetes and triglyceride levels. Science 316: 1331-6. doi: 1142358 [pii]
10.1126/science.1142358
Scott LJ, Mohlke KL, Bonnycastle LL, Willer CJ, Li Y, Duren WL, Erdos MR, Stringham HM, Chines PS, Jackson AU, Prokunina-Olsson L, Ding CJ, Swift AJ, Narisu N, Hu T, Pruim R, Xiao R, Li XY, Conneely KN, Riebow NL, Sprau AG, Tong M, White PP, Hetrick KN, Barnhart MW, Bark CW, Goldstein JL, Watkins L, Xiang F, Saramies J, Buchanan TA, Watanabe RM, Valle TT, Kinnunen L, Abecasis GR, Pugh EW, Doheny KF, Bergman RN, Tuomilehto J, Collins FS, Boehnke M (2007) A genome-wide association study of type 2 diabetes in Finns detects multiple susceptibility variants. Science 316: 1341-5. doi: 10.1126/science.1142382
Sladek R, Rocheleau G, Rung J, Dina C, Shen L, Serre D, Boutin P, Vincent D, Belisle A, Hadjadj S, Balkau B, Heude B, Charpentier G, Hudson TJ, Montpetit A, Pshezhetsky AV, Prentki M, Posner BI, Balding DJ, Meyre D, Polychronakos C, Froguel P (2007) A genome-wide association study identifies novel risk loci for type 2 diabetes. Nature 445: 881-5. doi: nature05616 [pii]
10.1038/nature05616
Takeuchi F, Serizawa M, Yamamoto K, Fujisawa T, Nakashima E, Ohnaka K, Ikegami H, Sugiyama T, Katsuya T, Miyagishi M, Nakashima N, Nawata H, Nakamura J, Kono S, Takayanagi R, Kato N (2009) Confirmation of multiple risk Loci and genetic impacts by a genome-wide association study of type 2 diabetes in the Japanese population. Diabetes 58: 1690-9. doi: 10.2337/db08-1494
Tsai FJ, Yang CF, Chen CC, Chuang LM, Lu CH, Chang CT, Wang TY, Chen RH, Shiu CF, Liu YM, Chang CC, Chen P, Chen CH, Fann CS, Chen YT, Wu JY (2010) A genome-wide association study identifies susceptibility variants for type 2 diabetes in Han Chinese. PLoS Genet 6: e1000847. doi: 10.1371/journal.pgen.1000847
Voight BF, Scott LJ, Steinthorsdottir V, Morris AP, Dina C, Welch RP, Zeggini E, Huth C, Aulchenko YS, Thorleifsson G, McCulloch LJ, Ferreira T, Grallert H, Amin N, Wu G, Willer CJ, Raychaudhuri S, McCarroll SA, Langenberg C, Hofmann OM, Dupuis J, Qi L, Segre AV, van Hoek M, Navarro P, Ardlie K, Balkau B, Benediktsson R, Bennett AJ, Blagieva R, Boerwinkle E, Bonnycastle LL, Bengtsson Bostrom K, Bravenboer B, Bumpstead S, Burtt NP, Charpentier G, Chines PS, Cornelis M, Couper DJ, Crawford G, Doney AS, Elliott KS, Elliott AL, Erdos MR, Fox CS, Franklin CS, Ganser M, Gieger C, Grarup N, Green T, Griffin S, Groves CJ, Guiducci C, Hadjadj S, Hassanali N, Herder C, Isomaa B, Jackson AU, Johnson PR, Jorgensen T, Kao WH, Klopp N, Kong A, Kraft P, Kuusisto J, Lauritzen T, Li M, Lieverse A, Lindgren CM, Lyssenko V, Marre M, Meitinger T, Midthjell K, Morken MA, Narisu N, Nilsson P, Owen KR, Payne F, Perry JR, Petersen AK, Platou C, Proenca C, Prokopenko I, Rathmann W, Rayner NW, Robertson NR, Rocheleau G, Roden M, Sampson MJ, Saxena R, Shields BM, Shrader P, Sigurdsson G, Sparso T, Strassburger K, Stringham HM, Sun Q, Swift AJ, Thorand B, et al. (2010) Twelve type 2 diabetes susceptibility loci identified through large-scale association analysis. Nat Genet 42: 579-89. doi: 10.1038/ng.609
Yasuda K, Miyake K, Horikawa Y, Hara K, Osawa H, Furuta H, Hirota Y, Mori H, Jonsson A, Sato Y, Yamagata K, Hinokio Y, Wang HY, Tanahashi T, Nakamura N, Oka Y, Iwasaki N, Iwamoto Y, Yamada Y, Seino Y, Maegawa H, Kashiwagi A, Takeda J, Maeda E, Shin HD, Cho YM, Park KS, Lee HK, Ng MC, Ma RC, So WY, Chan JC, Lyssenko V, Tuomi T, Nilsson P, Groop L, Kamatani N, Sekine A, Nakamura Y, Yamamoto K, Yoshida T, Tokunaga K, Itakura M, Makino H, Nanjo K, Kadowaki T, Kasuga M (2008) Variants in KCNQ1 are associated with susceptibility to type 2 diabetes mellitus. Nat Genet 40: 1092-7. doi: 10.1038/ng.207
Zabaneh D, Balding DJ (2010) A genome-wide association study of the metabolic syndrome in Indian Asian men. PLoS One 5: e11961. doi: 10.1371/journal.pone.0011961
Zeggini E, Scott LJ, Saxena R, Voight BF, Marchini JL, Hu T, de Bakker PI, Abecasis GR, Almgren P, Andersen G, Ardlie K, Bostrom KB, Bergman RN, Bonnycastle LL, Borch-Johnsen K, Burtt NP, Chen H, Chines PS, Daly MJ, Deodhar P, Ding CJ, Doney AS, Duren WL, Elliott KS, Erdos MR, Frayling TM, Freathy RM, Gianniny L, Grallert H, Grarup N, Groves CJ, Guiducci C, Hansen T,

Herder C, Hitman GA, Hughes TE, Isomaa B, Jackson AU, Jorgensen T, Kong A, Kubalanza K, Kuruvilla FG, Kuusisto J, Langenberg C, Lango H, Lauritzen T, Li Y, Lindgren CM, Lyssenko V, Marvelle AF, Meisinger C, Midthjell K, Mohlke KL, Morken MA, Morris AD, Narisu N, Nilsson P, Owen KR, Palmer CN, Payne F, Perry JR, Pettersen E, Platou C, Prokopenko I, Qi L, Qin L, Rayner NW, Rees M, Roix JJ, Sandbaek A, Shields B, Sjogren M, Steinthorsdottir V, Stringham HM, Swift AJ, Thorleifsson G, Thorsteinsdottir U, Timpson NJ, Tuomi T, Tuomilehto J, Walker M, Watanabe RM, Weedon MN, Willer CJ, Illig T, Hveem K, Hu FB, Laakso M, Stefansson K, Pedersen O, Wareham NJ, Barroso I, Hattersley AT, Collins FS, Groop L, McCarthy MI, Boehnke M, Altshuler D (2008) Meta-analysis of genome-wide association data and large-scale replication identifies additional susceptibility loci for type 2 diabetes. Nat Genet 40: 638-45. doi: ng.120 [pii]
10.1038/ng.120

Zeggini E, Weedon MN, Lindgren CM, Frayling TM, Elliott KS, Lango H, Timpson NJ, Perry JR, Rayner NW, Freathy RM, Barrett JC, Shields B, Morris AP, Ellard S, Groves CJ, Harries LW, Marchini JL, Owen KR, Knight B, Cardon LR, Walker M, Hitman GA, Morris AD, Doney AS, McCarthy MI, Hattersley AT (2007) Replication of genome-wide association signals in UK samples reveals risk loci for type 2 diabetes. Science 316: 1336-41. doi: 1142364 [pii]
10.1126/science.1142364
